# Crystal structures of the [Ir^III^{C(C_4_H_6_O_2_)(dppm)-κ^3^ *P*,*C*,*O*}(dppm)H](CF_3_O_3_S)_2_ and [Ir^III^{C(C_4_H_6_O_2_)(dppm)-κ^2^ *P*,*C*}(CO)(dppm)H](CF_3_O_3_S)_2_ phosphorus ylide complexes, generated by a Wittig-type carbon–carbon coupling reaction of a carbodiphospho­rane PCP ligand system

**DOI:** 10.1107/S205698901801455X

**Published:** 2018-10-23

**Authors:** Inge Schlapp-Hackl, Bettina Pauer, Christoph Falschlunger, Walter Schuh, Holger Kopacka, Klaus Wurst, Paul Peringer

**Affiliations:** a University of Innsbruck, Faculty of Chemistry and Pharmacy, Innrain 80-82, 6020 Innsbruck, Austria

**Keywords:** C=C coupling reaction, carbodi­phospho­rane (CDP), iridium(III), PCP pincer, ethyl diazo­acetate, crystal structure, NMR

## Abstract

The reaction of [Ir^III^{C(dppm)_2_-κ^3^ *P*,*C*,*P*′}ClH(NH_3_C_2_)]Cl with ethyl diazo­acetate, a well known C=C coupling reagent, leads to the formation of a C=C unit, accompanied by N_2_ abstraction, reorganization of a dppm subunit and, considered as a whole, to the transformation of the PCP pincer carbodi­phospho­rane system to a phospho­rus ylide ligand. After removal of the halogenides, the iridium center is stabilized by the carbonyl O atom through the formation of a five-membered chelate ring. A PCO pincer ligand system is thereby generated, which coordinates the iridium(III) atom threefold in a *facial* manner. The phospho­rus electron-donor atoms and the ylide carbon atom of the resulting [Ir^III^{C(C_4_H_6_O_2_)(dppm)-κ^3^ *P*,*C*,*O*}(dppm)H](CF_3_O_3_S)_2_ complex, also termed as [bis­(di­phenyl­phosphan­yl)methane]({[(di­phenyl­phosphan­yl)meth­yl]di­phenyl­phosphanyl­idene}(eth­oxy­oxoethanyl­idene)methanyl­idene-κ^3^ *P*,*C*,*O*)hydridoiridium(III) bis­(tri­fluoro­methane­sulfonate), are in plane and the hydrido ligand and the carbonyl O atom are located *trans* to each other, perpendicular to the *meridional* plane. The addition of carbon monoxide causes a replacement of the carbonyl O atom of the acetate subunit by a carbonyl ligand, thereby creating [bis­(di­phenyl­phosphan­yl)methane]­carbon­yl({[(di­phenyl­phosphan­yl)meth­yl]di­phenyl­phosphanyl­idene}(eth­oxy­oxoethanyl­idene)methanyl­idene-κ^2^ *P*,*C*}hydridoiridium(III) bis­(tri­fluoro­methane­sulfonate)–di­chloro­methane–ethyl acetate (6/2/3) or, more simply, [Ir^III^{C(C_4_H_6_O_2_)(dppm)-κ^2^ *P*,*C*}(CO)(dppm)H](CF_3_O_3_S)_2_·0.33CH_2_Cl_2_·0.5C_4_H_8_O_2_. One tri­fluoro­meth­ane­sulfonate counter-ion of **3** shows positional disorder in a 2:1 ratio. Complex **4** shows pseudo-merohedral twinning (matrix:  0 0 0  0 1 0 1). The di­chloro­methane solvent is disordered over two orientations with occupation factors of 0.5 and 0.166.

## Abstract 2

The reaction of [Ir^III^{C(dppm)_2_-κ^3^ *P*,*C*,*P*′}ClH(NH_3_C_2_)]Cl with ethyl diazo­acetate, a well known C=C coupling reagent, leads to the formation of a C=C unit, accompanied by N_2_ abstraction, and reorganization of a dppm subunit and, considered as a whole, to the transformation of the PCP pincer carbodi­phospho­rane system to a phospho­rus ylide ligand. After removal of the halogenides, the iridium center is stabilized by the carbonyl O atom through the formation of a five-membered chelate ring. A PCO pincer ligand system is thereby generated, which coordinates the iridium(III) atom threefold in a *facial* manner. The addition of carbon monoxide causes a replacement of the carbonyl O atom of the acetate subunit by a carbonyl ligand.

## Chemical context   

A divalent carbon(0) atom in an excited singlet (^1^ *D*) state, which may act as an electron-donor atom towards one or two Lewis acids, opens up a wide range of functionalities and chemical properties (Petz & Frenking, 2010 ▸). We decided to investigate these intriguing properties in detail and to explore this unusual donor species, generally known as a carbodi­phospho­rane (CDP) carbon atom, in combination with the transition metal iridium. We had earlier designed a new and innovative PCP pincer ligand system, which allows the stabil­ization of a carbodi­phospho­rane atom by two dppm subunits or, more precisely, by two tertiary phosphines *via* donor–acceptor inter­actions (Stallinger *et al.*, 2007 ▸). The central carbon atom exhibits two lone electron pairs and can also be referred to as a phospho­rus double ylide.
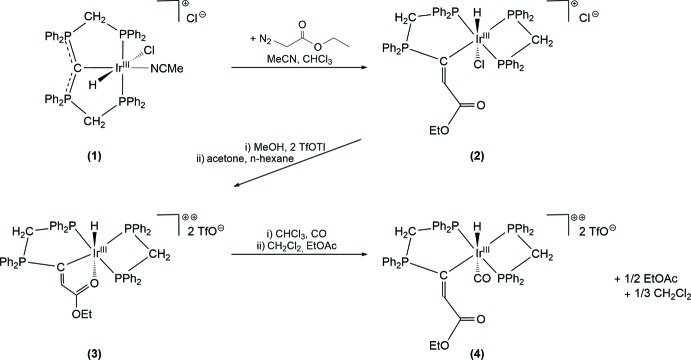


Treatment of our PCP pincer ligand system [CH(dppm)_2_]Cl (Stallinger *et al.*, 2007 ▸) with [Ir^I^Cl(cod)]_2_ causes a threefold coordination of the iridium(I) transition metal, a deprotonation of the carbodi­phospho­rane carbon atom, followed by a protonation and a subsequent oxidation of the iridium(I) center. Addition of ethyl diazo­acetate (EDA) to [Ir{C(dppm)_2_-κ^3^ *P*,*C*,*P*′}ClH(MeCN)]Cl complex **1** (Scheme 1) leads to a carbon–carbon coupling reaction *via* extrusion of a dppm subunit, which is stabilized by two phospho­rus–iridium(III) electron donor–acceptor inter­actions under the formation of a four-membered chelate ring. This reaction sequence, produced by the inter­action of the doubly ylidic carbon atom with an electrophile containing the extraordinarily good di­nitro­gen withdrawing group, may be described as Wittig-type carbon–carbon coupling reaction, which has rarely been reported in carbodi­phospho­rane chemistry (Kolodiazhnyi, 1999 ▸; Petz & Frenking, 2010 ▸). Furthermore, in analogy to Schmidbaur (1983 ▸), a specification of the process as a substitution reaction, during which one phosphine is replaced by a carbene ligand, is possible. The alkyl­idene C(dppm) unit coordinates the iridium(III) metal center in a *κ^2^P,C* manner. Overall, the carbodi­phospho­rane has been converted into a phospho­rus ylide ligand. Perpendicularly located to the dppm and C(dppm) units, the iridium(III) center coordinates a hydrido and a chlorido ligand *trans* to each other. Reaction of the monocationic [Ir{C(C_4_H_6_O_2_)(dppm)-*κ^2^P,C*}Cl(dppm)H]Cl complex **2** with two equivalents of thallium(I)tri­fluoro­methane­sulfonate (TfOTl) causes the removal of the chlorido ligand and the chloride counter-ion with concomitant coordination of the acetate carbonyl oxygen atom in a *facial* manner, resulting in formation of the dicationic [Ir{C(C_4_H_6_O_2_)(dppm)-*κ^3^P,C,O*}(dppm)H](CF_3_O_3_S)_2_ complex **3**.

A similar ligand arrangement in the coordination sphere of manganese(I) was previously mentioned by Ruiz *et al.* (2005 ▸). Protonation of the *σ*-alkynyl functionality of the [Mn^I^(C≡C—CO_2_Me)(CO)_3_(dppm)] complex at low temperature generates the corresponding vinyl­idene [Mn^I^(C=CH—CO_2_Me)(CO)_3_(dppm)]BF_4_ complex, which rearranges *via* insertion of the vinyl­idene ligand into the manganese–phospho­rus bond upon warming to room temperature to an [Mn^I^{(dppm)C=CH(CO_2_Me)}(CO)_3_]BF_4_ complex. Exposure of complex **3** to carbon monoxide gas cleaves the iridium(III) carbonyl oxygen bond under coordination of a carbonyl ligand. Up to now, we have been unable to obtain suitable single crystals of complexes **1** and **2**; however, it proved possible to crystallize the [Ir{C(C_4_H_6_O_2_)(dppm)-*κ^3^P,C,O*}(dppm)H](CF_3_O_3_S)_2_ (**3**) and [Ir{C(C_4_H_6_O_2_)(dppm)-*κ* ^2^ *P,C*}(CO)(dppm)H](CF_3_O_3_S)_2_ (**4**) products, the latter as a mixed di­chloro­methane–ethyl acetate solvate.

## Structural commentary   

**Figure 1:**
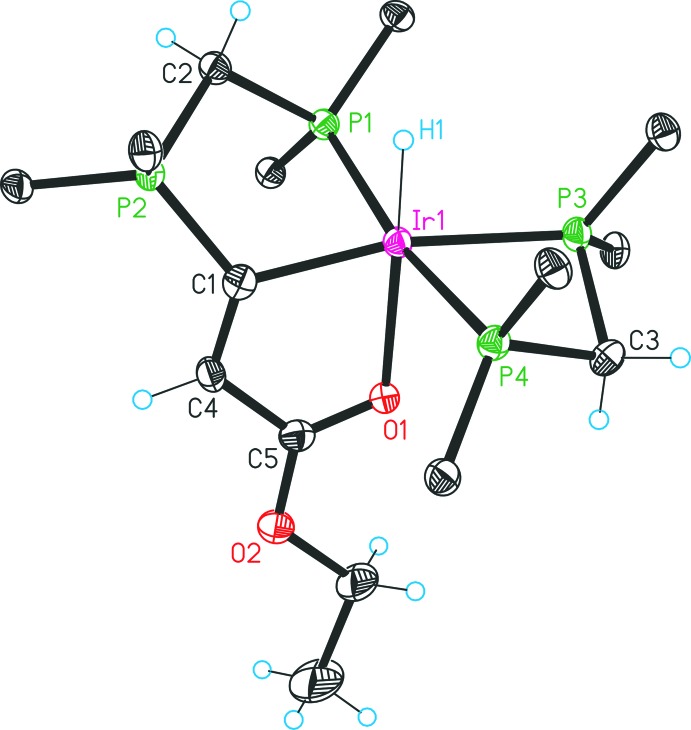
Structure of complex **3** with displacement ellipsoids drawn at the 30% probability level. For clarity, only the *ipso* carbon atoms of the phenyl groups are shown and the counter-ions are omitted.

The single crystal data for **3** reveal an ortho­rhom­bic crystal system in space group *P*2_1_2_1_2_1_ (Fig. 1 ▸). The complex can be described as an asymmetric dicationic Ir^III^ complex, stabilized by two tri­fluoro­methane­sulfonate counter-ions. The iridium(III) centre is coordinated in a *facial* mode by the PCO pincer ligand system *via* a phosphine functionality, an ylidic carbon atom and a carbonyl oxygen atom of the ester group. The coordination sphere of the iridium(III) atom is completed by one bidentate dppm and one hydrido ligand. Furthermore, all phospho­rus atoms and the iridium atom are positioned in a common plane and the carbonyl oxygen atom as well as the hydride anion are located perpendicularly to this plane, *trans* to each other. The iridium center creates with its coordination sphere a distorted octa­hedral geometry and the deviations are caused by the presence of the strained four-membered dppm chelate ring [P3—Ir1—P4 = 70.2 (1)°] and the tridentate PCO ligand [C1—Ir1—O1 = 75.5 (2)°; C1—Ir1—P1 = 79.45 (17)°; O1—Ir1—P1 = 109.2 (1)°]. A C1—C4 distance of 1.335 (9) Å indicates a double bond and the sum of angles of 358.3° [C4—C1—P2 = 124.8 (5)°; C4—C1—Ir1 = 118.0 (5)°; P2—C1—Ir1 = 115.5 (3) °] permits the designation of the C1 surroundings as a planar surface. The Ir1—O1 bond length of 2.239 (4) Å is close to the value [2.262 (4) Å] in the related [Ir(CO_2_CH_3_){C(CO_2_CH_3_)CH(CO_2_CH_3_)}(PPh_3_)(2-Ph_2_PC_6_H_4_NH)] complex (Dahlenburg & Herbst, 1999 ▸).

**Figure 2:**
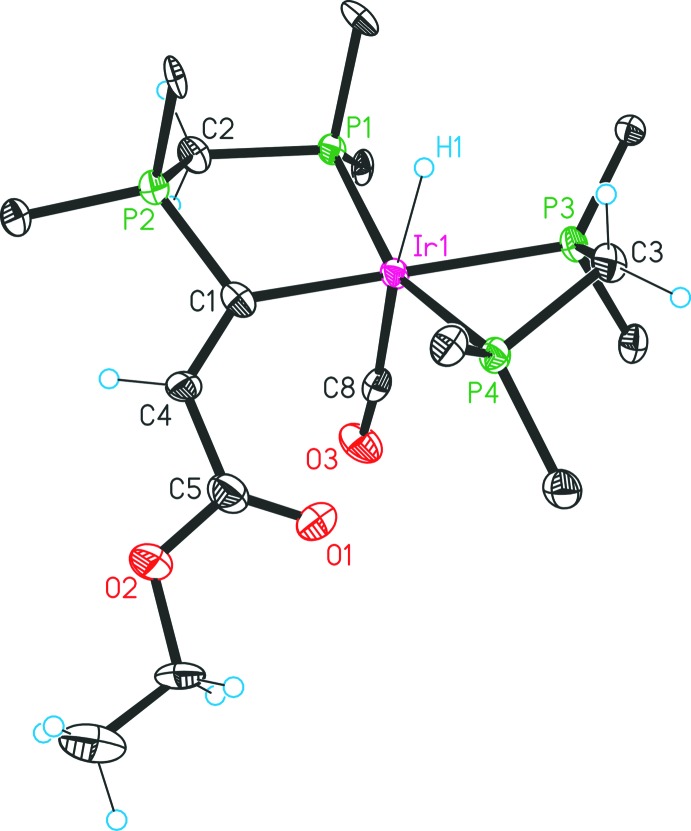
Structure of one of the two independent units in complex **4** with displacement ellipsoids drawn at the 30% probability level. For clarity, only the *ipso* carbon atoms of the phenyl groups are shown and the counter-ions and solvent mol­ecules are omitted.

**Figure 3:**
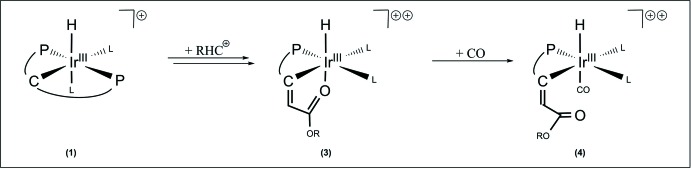
The sequence of ligand replacements and reorganizations accompanying the transformation of **2** into **3** and then **4**.

The solvated complex **4** crystallizes in the monoclinic space group *P*2_1_/*c* and each asymmetric unit contains two closely related formula units. Complex **4** can be described as a bulky dicationic iridium(III) complex, which is stabilized by two tri­fluoro­methane­sulfonate counter-ions (Fig. 2 ▸). In comparison with complex **3**, many structural characteristics are similar. The iridium(III) metal atom shows a distorted octa­hedral geometry. It coordinates a dppm unit and a PCO pincer ligand system in a *meridional* manner and, perpendicular to this plane, a hydrido ligand. The only difference is that the carbonyl oxygen atom of the PCO ligand system is uncoordinated and has been substituted by a carbonyl ligand. The carbonyl ligand reveals relatively long Ir1—C8 [1.965 (15) Å] and C8—O3 [1.116 (14) Å] distances, caused by the location *trans* to the hydrido ligand. Moreover, the substitution results in an overall lengthening of the Ir—P and the Ir—C separations (Table 1 ▸). This effect is especially pronounced for the Ir1—C1 value, which rises by an amount of 0.07 Å. Additionally, the substitution causes an approximation of the C1—Ir1—P1 angle [88.5 (3)°] to a regular octa­hedral angle of about 90° and an increase of the C4—C1—Ir1 angle to a value of 134.0 (9)°. Notably, the coordination of the carbonyl functionality has almost no effect on the C1—C4 double bond (Table 1 ▸) and considered in total the planar environment of the C1 atom [sum of the angles (C4—C1—P2; C4—C1—Ir1; P2—C1—Ir1) = 359.2°] is barely affected. The sequence of ligand replacements and reorganizations for **2**, **3** and **4** are shown in Fig. 3 ▸.

## Supra­molecular features   

In the crystal of **3**, the counter-ions inter­act with the hydrido ligand and with the hydrogen atoms of the dppm methyl­ene groups, leading to C⋯O and C⋯F separations of between 3.188 (11) and 3.473 (10) Å (Table 2 ▸). Such inter­actions are well known in connection with dppm and related ligand systems (Jones & Ahrens, 1998 ▸).

In the extended structure of **4**, the complex shows additional inter­actions between the tri­fluoro­methane­sulfonate counter-ions and the hydrido ligand and the hydrogen atoms of the dppm methyl­ene groups, respectively (Table 3 ▸).

## Synthesis and crystallization   

The [CH(dppm)_2_]Cl compound was prepared by a previously reported procedure (Reitsamer *et al.*, 2012 ▸); other starting materials and solvents were obtained from commercial suppliers. All preparations were carried out under an inert gas atmosphere of di­nitro­gen by the use of standard Schlenk techniques. The ^1^H, ^13^C and ^31^P NMR spectra were recorded on a Bruker DPX 300 NMR spectrometer and were referenced against the solvent peaks of di­chloro­methane, chloro­form or aceto­nitrile, respectively, or, in the case of the ^31^P nucleus, against an external aqueous 85% H_3_PO_4_ standard. The phospho­rus atoms in the NMR data are labelled as in Figs. 1 ▸ and 2 ▸.

**Synthesis of [Ir{C(dppm)_2_-** ***κ^3^P,C,P***′**}ClH(MeCN)]Cl (1):** A mixture of 0.1 ml MeCN, 20.4 mg of [CH(dppm)_2_]Cl (0.0250 mmol) and 8.4 mg of [IrCl(cod)]_2_ (0.0125 mmol) was stirred for 1 min. The resulting solution contains predominantly the well known [Ir{CH(dppm)_2_-*κ^3^P,C,P*′}(cod)]Cl_2_ (Partl *et al.*, 2018 ▸) and minor amounts of the [Ir{C(dppm)_2_-*κ^3^P,C,P*′}ClH(MeCN)]Cl complex **1**. ^31^P {^1^H} NMR (CH_2_Cl_2_/C_2_H_3_N, 5:1): δ = 1.5 (*vt*, P1/P4, N = 70.4 Hz), 31.5 (*vt*, P2/P3) p.p.m.; ^13^C {^1^H} NMR (CD_2_Cl_2_ / C_2_D_3_N, 5:1): δ = −28.9 (*t*, C1, ^1^ *J*_P2/P3C1_ = 99.6 Hz) p.p.m.; ^1^H NMR (CDCl_3_/C_2_D_3_N, 5:1): δ = −21.3 (*t*, hydride, ^2^ *J*_PH_ = 13.2 Hz) p.p.m.

**Synthesis of [Ir{C(C_4_H_6_O_2_)(dppm)-** ***κ^2^P,C*** **}Cl(dppm)H]Cl (2):** While stirring the aforementioned MeCN solution of [Ir{CH(dppm)_2_-*κ^3^P,C,P*′}(cod)]Cl_2_ and [Ir{C(dppm)_2_-*κ^3^P,C,P*′}ClH(MeCN)]Cl (**1**), 0.26 ml of CHCl_3_ and 0.24 ml of a solution of ethyl diazo­acetate in CHCl_3_ (*c* = 0.105 mol l^−1^; 0.025 mmol) were added successively. The [Ir{CH(dppm)_2_-*κ^3^P,C,P*′}(cod)]Cl_2_ by-product is slowly transformed to **1**, which in turn reacts with ethyl diazo­acetate. After standing for 24 h, product **2** was generated almost qu­anti­tatively. ^31^P {^1^H} NMR (CHCl_3_/C_2_H_3_N, 5:1): δ = 26.5 (*ddd*, P1, ^2^ *J*_P1P2_ = 52.1 Hz, ^2^ *J*_P1P3_ = 16.8 Hz, ^2^ *J*_P1P4_ = 371.1 Hz), 45.7 (*ddd*, P2, ^3^ *J*_P2P3_ = 18.4 Hz, ^4^ *J*_P2P4_ = 7.3 Hz), −52.5 (*ddd*, P3, ^2^ *J*_P4P3_ = 30.6 Hz), −36.7 (*ddd*, P4) p.p.m.; ^13^C {^1^H} NMR (CDCl_3_/C_2_D_3_N, 5:1): δ = 139.2 (*dddd*, C1, ^2^ *J*_P1C1_ = 6.9 Hz, ^1^ *J*_P2C1_ = 6.9 Hz, ^2^ *J*_P3C1_ = 6.9 Hz, ^2^ *J*_P4C1_ = 98.2 Hz) p.p.m.; ^1^H NMR (CDCl_3_/C_2_D_3_N, 5:1): δ = −17.7 (*ddd*, hydride, ^2^ *J*_P1H_ = 9.9 Hz, ^2^ *J*_P3H_ = 9.9 Hz, ^2^ *J*_P4H_ = 9.9 Hz) p.p.m.

**Synthesis of [Ir{C(C_4_H_6_O_2_)(dppm)-** ***κ^3^P,C,O*** **}(dppm)H](CF_3_O_3_S)_2_ (3):** 21.2 mg of thallium(I) tri­fluoro­methane­sulfonate (0.0597 mmol) were dissolved in MeOH (0.1 ml) and added to a solution of complex **2** (0.025 mmol) in chloro­form/aceto­nitrile (5:1). After stirring for 15 min the precipitated TlCl was separated, all solvents were removed and complex **3** was obtained (34.0 mg, 100%). Single beige–white crystals of complex **3** were grown slowly from acetone (0.5 ml), covered with 0.3 ml of hexane. ^31^P {^1^H} NMR (CHCl_3_/C_2_H_3_N, 5:1): δ = 9.1 (*ddd*, P1, ^2^ *J*_P1P2_ = 27.6 Hz, ^2^ *J*_P1P3_ = 13.0 Hz, ^2^ *J*_P1P4_ = 311.2 Hz), 36.0 (*ddd*, P2, ^3^ *J*_P2P3_ = 13.8 Hz), −44.2 (*ddd*, P3, ^2^ *J*_P3P4_ = 35.2 Hz), −33.1 (*dd*, P4) p.p.m.; ^13^C {^1^H} NMR (CDCl_3_): δ = 181.4 (*dddd*, C1, ^3^ *J*_P1C1_ = 6.1 Hz, ^1^ *J*_P2C1_ = 34.8 Hz, ^2^ *J*_P3C1_ = 81.2 Hz, ^2^ *J*_P4C1_ = 2.8 Hz) p.p.m.; ^1^H NMR (CDCl_3_): δ = −23.3 (*ddd*, hydride, ^2^ *J*_PH_ = 6.7 Hz, ^2^ *J*_PH_ = 12.1 Hz, ^2^ *J*_PH_ = 20.8 Hz) p.p.m.

**Synthesis of [Ir{C(C_4_H_6_O_2_)(dppm)-** ***κ*** **^2^** ***P,C*** **}(CO)(dppm)H](CF_3_O_3_S)_2_ (4):** Complex **3** was dissolved in CHCl_3_ (0.6 ml), the mixture was filtered and the solution was placed in an atmos­phere of CO. After standing for 16 h product **4** was formed and single crystals were obtained *via* layering of a solution of complex **4** dissolved in CH_2_Cl_2_ with EtOAc. ^31^P {^1^H} NMR (CHCl_3_/CH_3_OH): δ = 5.3 (*ddd*, P1, ^2^ *J*_P1P2_ = 50.3 Hz, ^2^ *J*_P1P3_ = 16.9 Hz, ^2^ *J*_P1P4_ = 296.0 Hz), 57.1 (*ddd*, P2, ^3^ *J*_P2P3_ = 15.3 Hz, ^3^ *J*_P2P4_ = 12.3 Hz), −55.6 (*ddd*, P3, ^2^ *J*_P3P4_ = 32.8 Hz), −44. 7 (*ddd*, P4) p.p.m.; ^13^C {^1^H} NMR (CDCl_3_/CD_3_OD): δ = 134.8 (*ddd*, C1, ^1^ *J*_C1P2_ = 6.9 Hz, ^2^ *J*_C1P3_ = 98.2 Hz) p.p.m.; ^1^H NMR (CDCl_3_/CD_3_OD): δ = −8.8 (*t*, hydride, ^2^ *J*_PH_ = 13.5 Hz) p.p.m.

## Refinement   

Crystal data, data collection and structure refinement details are summarized in Table 4 ▸. The data for both **3** and **4** were processed without absorption corrections. In relation to the structure determination of complex **3**, the hydrido ligand was detected and refined isotropically. One tri­fluoro­methane­sulfonate counter-ion shows positional disorder in a 2:1 ratio, caused by an overlying of the C9, O16 and F16 positions. These positions were also refined isotropically. The structure determination of complex **4** resulted in the detection of pseudo-merohedral twinning (matrix: 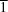 0 0 0 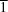 0 1 0 1). Furthermore, the hydrido ligand was determined and refined isotropically by the use of a bond restraint of 1.6 Å and a fixed *U*_iso_ value. The solvent di­chloro­methane shows disorder over two orientations, which can be described with occupation factors 0.5 and 0.166. Refinement of this solvent mol­ecule was carried out by the usage of bond restraints and isotropic displacement parameters. Furthermore, the ethyl acetate mol­ecule was located and modelled with equal anisotropic displacement parameters for O21, C22 and C23. H atoms bound to Ir1 and C4 were located in a difference-Fourier map and refined isotropically. Other H atoms were positioned geometrically and refined using a riding model with C—H = 0.94–0.98 Å and *U*_iso_(H) = 1.2*U*_eq_(C).

## Supplementary Material

Crystal structure: contains datablock(s) global, 3, 4. DOI: 10.1107/S205698901801455X/hb7757sup1.cif

Structure factors: contains datablock(s) 3. DOI: 10.1107/S205698901801455X/hb77573sup2.hkl

Structure factors: contains datablock(s) 4. DOI: 10.1107/S205698901801455X/hb77574sup4.hkl

CCDC references: 1849369, 1849368

Additional supporting information:  crystallographic information; 3D view; checkCIF report

## Figures and Tables

**Table 1:** Selected distances and angles (Å, °) in complexes **3** and **4**

	Complex **3**	Complex **4**
Ir1—C1	2.062 (6)	2.131 (11)
Ir1—P1	2.295 (2)	2.334 (3)
Ir1—P3	2.333 (2)	2.379 (3)
Ir1—P4	2.341 (2)	2.377 (3)
Ir1—H1	1.58 (5)	1.60 (2)
C1—C4	1.335 (9)	1.342 (15)
C4—C5	1.460 (9)	1.461 (16)
P2—C1	1.789 (7)	1.826 (12)
O1—C5	1.248 (8)	1.190 (14)
C1—Ir1—P1	79.5 (2)	88.5 (3)
P4—Ir1—P3	70.2 (1)	69.3 (1)

**Table 2:** Hydrogen-bond geometry (Å, °) for **3** [Chem scheme1]

*D*—H⋯*A*	*D*—H	H⋯*A*	*D*⋯*A*	*D*—H⋯*A*
C2—H2*A*⋯O11	0.98	2.53	3.420 (9)	151
C3—H3*A*⋯F15*A* ^i^	0.98	2.48	3.310 (19)	142
C6—H6*A*⋯O14*A* ^i^	0.98	2.55	3.34 (3)	138
C6—H6*B*⋯O15*A*	0.98	2.38	3.32 (3)	160
C106—H106⋯O11	0.94	2.39	3.293 (10)	162
C108—H108⋯O1	0.94	2.43	3.255 (8)	147
C112—H112⋯O12^ii^	0.94	2.55	3.164 (9)	123
C206—H206⋯O11	0.94	2.61	3.473 (10)	152
C212—H212⋯O13^iii^	0.94	2.55	3.220 (9)	129
C306—H306⋯O1	0.94	2.61	3.396 (8)	141
C312—H312⋯O13^iv^	0.94	2.51	3.298 (9)	141
C406—H406⋯O16^i^	0.94	2.63	3.188 (11)	119

Symmetry codes: (i) 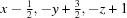; (ii) 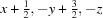; (iii) 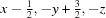; (iv) 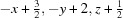.

**Table 3:** Hydrogen-bond geometry (Å, °) for **4** [Chem scheme1]

*D*—H⋯*A*	*D*—H	H⋯*A*	*D*⋯*A*	*D*—H⋯*A*
C2—H2*A*⋯O43	0.98	2.23	3.190 (15)	165
C3—H3*B*⋯O41^i^	0.98	2.44	3.404 (14)	167
C12—H12*B*⋯O25^i^	0.98	2.26	3.226 (14)	170
C13—H13*A*⋯O38^ii^	0.98	2.58	3.538 (15)	167
C13—H13*B*⋯O26	0.98	2.48	3.446 (15)	169
C16—H16*B*⋯O20^iii^	0.98	2.48	3.15 (2)	125
C102—H102⋯O39	0.94	2.47	3.379 (17)	162
C108—H108⋯O11^i^	0.94	2.57	3.248 (15)	129
C202—H202⋯O39	0.94	2.57	3.508 (16)	178
C212—H212⋯O43	0.94	2.55	3.471 (15)	167
C306—H306⋯O11^i^	0.94	2.53	3.409 (16)	155
C312—H312⋯O41^i^	0.94	2.46	3.374 (16)	166
C402—H402⋯O11^i^	0.94	2.52	3.429 (15)	164
C506—H506⋯O12^i^	0.94	2.50	3.420 (17)	165
C508—H508⋯O38^ii^	0.94	2.56	3.284 (16)	134
C602—H602⋯O12^i^	0.94	2.59	3.519 (16)	172
C612—H612⋯O25^i^	0.94	2.59	3.511 (17)	165
C706—H706⋯O38^ii^	0.94	2.47	3.327 (16)	151
C712—H712⋯O26	0.94	2.41	3.335 (18)	167
C806—H806⋯O38^ii^	0.94	2.52	3.406 (16)	158
C808—H808⋯O4	0.94	2.48	2.964 (16)	112
C25—H25*B*⋯O24	0.98	2.53	3.14 (3)	121
C25*A*—H25*D*⋯O24	0.98	2.19	3.10 (9)	153
C16—H16*B*⋯O20^iii^	0.98	2.48	3.15 (2)	125

Symmetry codes: (i) 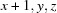; (ii) 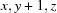; (iii) 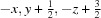.

**Table 4:** Experimental details

	**3**	**4**
Crystal data		
Chemical formula	[IrH(C_30_H_28_O_2_P_2_)(C_25_H_22_P_2_)](CF_3_O_3_S)_2_	[IrH(C_30_H_28_O_2_P_2_)(C_25_H_22_P_2_)(CO)](CF_3_O_3_S)_2_·0.33CH_2_Cl_2_·0.5C_4_H_8_O_2_
*M*_r_	1358.17	1458.54
Crystal system, space group	Orthorhombic, *P*2_1_2_1_2_1_	Monoclinic, *P*2_1_/*c*
Temperature (K)	233	233
*a*, *b*, *c* (Å)	11.3249 (1), 21.3629 (3), 23.4125 (3)	14.4712 (3), 20.9611 (5), 41.3651 (9)
α, β, γ (°)	90, 90, 90	90, 100.108 (1), 90
*V* (Å^3^)	5664.25 (12)	12352.6 (5)
*Z*	4	8
Radiation type	Mo *K*α	Mo *K*α
μ (mm^−1^)	2.62	2.44
Crystal size (mm)	0.41 × 0.05 × 0.04	0.15 × 0.09 × 0.04

Data collection		
Diffractometer	Nonius KappaCCD	Nonius KapppCCD
No. of measured, independent and observed [*I* > 2σ(*I*)] reflections	36212, 9983, 9331	45373, 18805, 14603
*R*_int_	0.046	0.068
θ_max_ (°)	25.0	24.0
(sin θ/λ)_max_ (Å^−1^)	0.595	0.572

Refinement		
*R*[*F* ^2^ > 2σ(*F* ^2^)], *wR*(*F* ^2^), *S*	0.029, 0.062, 1.03	0.054, 0.108, 1.04
No. of reflections	9983	18805
No. of parameters	701	1530
No. of restraints	0	5
H-atom treatment	H atoms treated by a mixture of independent and constrained refinement	H atoms treated by a mixture of independent and constrained refinement
Δρ_max_, Δρ_min_ (e Å^−3^)	0.62, −0.62	1.90, −0.93
Absolute structure	Flack *x* determined using 3851 quotients [(*I* ^+^)−(*I* ^−^)]/[(*I* ^+^)+(*I* ^−^)] (Parsons *et al.*, 2013 ▸)	–
Absolute structure parameter	−0.006 (2)	–

Computer programs: *COLLECT* (Nonius, 1999 ▸), *DENZO* and *SCALEPACK* (Otwinowski & Minor, 1997 ▸), *XP* in *SHELXTL* and *SHELXS97* (Sheldrick, 2008 ▸), *SHELXL2014/7* (Sheldrick, 2015 ▸) and *ChemDraw* (Cambridge Soft, 2001 ▸).

## supplementary crystallographic information

### [Bis(diphenylphosphanyl)methane]({[(diphenylphosphanyl)methyl]diphenylphosphanylidene}(ethoxyoxoethanylidene)methanylidene-κ3P,C,O)hydridoiridium(III) bis(trifluoromethanesulfonate) (3) Crystal data

**Table:**

[IrH(C_30_H_28_O_2_P_2_)(C_25_H_22_P_2_)](CF_3_O_3_S)_2_	*D*_x_ = 1.593 Mg m^−^^3^
*M**_r_* = 1358.17	Mo *K*α radiation, λ = 0.71073 Å
Orthorhombic, *P*2_1_2_1_2_1_	Cell parameters from 83772 reflections
*a* = 11.3249 (1) Å	θ = 1.0–25.3°
*b* = 21.3629 (3) Å	µ = 2.62 mm^−^^1^
*c* = 23.4125 (3) Å	*T* = 233 K
*V* = 5664.25 (12) Å^3^	Prism, colorless
*Z* = 4	0.41 × 0.05 × 0.04 mm
*F*(000) = 2720	

### [Bis(diphenylphosphanyl)methane]({[(diphenylphosphanyl)methyl]diphenylphosphanylidene}(ethoxyoxoethanylidene)methanylidene-κ3P,C,O)hydridoiridium(III) bis(trifluoromethanesulfonate) (3) Data collection

**Table:**

Nonius KappaCCD diffractometer	*R*_int_ = 0.046
Detector resolution: 9.4 pixels mm^-1^	θ_max_ = 25.0°, θ_min_ = 1.9°
phi– and ω–scans	*h* = −13→13
36212 measured reflections	*k* = −25→25
9983 independent reflections	*l* = −27→27
9331 reflections with *I* > 2σ(*I*)	

### [Bis(diphenylphosphanyl)methane]({[(diphenylphosphanyl)methyl]diphenylphosphanylidene}(ethoxyoxoethanylidene)methanylidene-κ3P,C,O)hydridoiridium(III) bis(trifluoromethanesulfonate) (3) Refinement

**Table:**

Refinement on *F*^2^	Hydrogen site location: mixed
Least-squares matrix: full	H atoms treated by a mixture of independent and constrained refinement
*R*[*F*^2^ > 2σ(*F*^2^)] = 0.029	*w* = 1/[σ^2^(*F*_o_^2^) + (0.0255*P*)^2^ + 6.1921*P*] where *P* = (*F*_o_^2^ + 2*F*_c_^2^)/3
*wR*(*F*^2^) = 0.062	(Δ/σ)_max_ = 0.001
*S* = 1.03	Δρ_max_ = 0.62 e Å^−^^3^
9983 reflections	Δρ_min_ = −0.62 e Å^−^^3^
701 parameters	Absolute structure: Flack *x* determined using 3851 quotients [(*I*^+^)-(*I*^-^)]/[(*I*^+^)+(*I*^-^)] (Parsons et al. (2013)
0 restraints	Absolute structure parameter: −0.006 (2)
Primary atom site location: structure-invariant direct methods	

### [Bis(diphenylphosphanyl)methane]({[(diphenylphosphanyl)methyl]diphenylphosphanylidene}(ethoxyoxoethanylidene)methanylidene-κ3P,C,O)hydridoiridium(III) bis(trifluoromethanesulfonate) (3) Special details

**Table:**

Geometry. All esds (except the esd in the dihedral angle between two l.s. planes) are estimated using the full covariance matrix. The cell esds are taken into account individually in the estimation of esds in distances, angles and torsion angles; correlations between esds in cell parameters are only used when they are defined by crystal symmetry. An approximate (isotropic) treatment of cell esds is used for estimating esds involving l.s. planes.
Refinement. Hydrogen atoms at C4 and Ir1 were found and isotropically refined. One trifat-anion is positional disordered in ratio 2:1 with overlying position for C9, O16 and F16. C, F and O atoms of this triflate were isotropically refined.

### [Bis(diphenylphosphanyl)methane]({[(diphenylphosphanyl)methyl]diphenylphosphanylidene}(ethoxyoxoethanylidene)methanylidene-κ3P,C,O)hydridoiridium(III) bis(trifluoromethanesulfonate) (3) Fractional atomic coordinates and isotropic or equivalent isotropic displacement parameters (Å^2^)

**Table:**

	*x*	*y*	*z*	*U*_iso_*/*U*_eq_	Occ. (<1)
Ir1	0.63504 (2)	0.81953 (2)	0.23353 (2)	0.02510 (7)	
H1	0.611 (4)	0.860 (2)	0.178 (2)	0.012 (13)*	
P1	0.79351 (13)	0.78749 (8)	0.17992 (7)	0.0268 (3)	
P2	0.58479 (14)	0.72005 (8)	0.13082 (7)	0.0285 (4)	
P3	0.69827 (14)	0.90758 (8)	0.28392 (7)	0.0291 (4)	
P4	0.47274 (13)	0.86937 (8)	0.27555 (7)	0.0289 (4)	
O1	0.6254 (4)	0.76165 (18)	0.31314 (16)	0.0290 (9)	
O2	0.5550 (4)	0.6701 (2)	0.34468 (19)	0.0448 (12)	
C1	0.5760 (5)	0.7333 (3)	0.2061 (3)	0.0300 (14)	
C2	0.7215 (5)	0.7609 (3)	0.1132 (3)	0.0292 (14)	
H2A	0.7043	0.7970	0.0887	0.035*	
H2B	0.7745	0.7327	0.0923	0.035*	
C3	0.5662 (5)	0.9072 (3)	0.3296 (3)	0.0333 (15)	
H3A	0.5761	0.8817	0.3641	0.040*	
H3B	0.5392	0.9493	0.3397	0.040*	
C4	0.5603 (5)	0.6886 (3)	0.2453 (3)	0.0313 (14)	
H4	0.541 (5)	0.646 (3)	0.239 (3)	0.038 (18)*	
C5	0.5814 (6)	0.7091 (3)	0.3039 (3)	0.0342 (15)	
C6	0.5818 (8)	0.6890 (4)	0.4033 (3)	0.059 (2)	
H6A	0.5427	0.7288	0.4122	0.071*	
H6B	0.6671	0.6945	0.4082	0.071*	
C7	0.5376 (12)	0.6386 (5)	0.4419 (4)	0.103 (4)	
H7A	0.5540	0.6497	0.4813	0.155*	
H7B	0.4531	0.6337	0.4367	0.155*	
H7C	0.5770	0.5995	0.4327	0.155*	
C101	0.8999 (5)	0.8454 (3)	0.1569 (3)	0.0315 (15)	
C102	0.9816 (6)	0.8656 (4)	0.1963 (3)	0.0429 (18)	
H102	0.9852	0.8465	0.2325	0.051*	
C103	1.0591 (7)	0.9143 (4)	0.1831 (4)	0.056 (2)	
H103	1.1157	0.9276	0.2099	0.067*	
C104	1.0518 (7)	0.9426 (4)	0.1308 (4)	0.065 (3)	
H104	1.1006	0.9770	0.1224	0.078*	
C105	0.9754 (8)	0.9215 (5)	0.0914 (4)	0.074 (3)	
H105	0.9738	0.9402	0.0550	0.089*	
C106	0.8986 (6)	0.8726 (4)	0.1034 (3)	0.056 (2)	
H106	0.8462	0.8580	0.0753	0.067*	
C107	0.8796 (5)	0.7219 (3)	0.2060 (3)	0.0287 (13)	
C108	0.8719 (6)	0.7053 (3)	0.2634 (3)	0.0335 (13)	
H108	0.8243	0.7290	0.2882	0.040*	
C109	0.9339 (6)	0.6541 (3)	0.2842 (3)	0.0381 (17)	
H109	0.9290	0.6434	0.3231	0.046*	
C110	1.0024 (6)	0.6191 (3)	0.2479 (3)	0.0420 (18)	
H110	1.0418	0.5835	0.2618	0.050*	
C111	1.0140 (6)	0.6356 (3)	0.1916 (3)	0.0421 (18)	
H111	1.0629	0.6120	0.1673	0.050*	
C112	0.9538 (5)	0.6871 (3)	0.1705 (3)	0.0352 (15)	
H112	0.9628	0.6986	0.1320	0.042*	
C201	0.4563 (6)	0.7559 (3)	0.1002 (3)	0.0327 (16)	
C202	0.3499 (6)	0.7497 (4)	0.1292 (3)	0.0487 (18)	
H202	0.3469	0.7276	0.1638	0.058*	
C203	0.2479 (7)	0.7763 (4)	0.1067 (4)	0.057 (2)	
H203	0.1753	0.7715	0.1257	0.068*	
C204	0.2540 (7)	0.8093 (4)	0.0573 (4)	0.057 (2)	
H204	0.1853	0.8281	0.0427	0.069*	
C205	0.3576 (7)	0.8156 (4)	0.0284 (3)	0.060 (2)	
H205	0.3594	0.8387	−0.0057	0.072*	
C206	0.4612 (7)	0.7883 (3)	0.0488 (3)	0.0427 (18)	
H206	0.5323	0.7917	0.0283	0.051*	
C207	0.5968 (6)	0.6404 (3)	0.1068 (3)	0.0336 (15)	
C208	0.7066 (7)	0.6107 (3)	0.1049 (3)	0.0445 (19)	
H208	0.7745	0.6315	0.1181	0.053*	
C209	0.7149 (7)	0.5504 (4)	0.0836 (3)	0.053 (2)	
H209	0.7893	0.5311	0.0815	0.063*	
C210	0.6176 (8)	0.5183 (3)	0.0656 (3)	0.052 (2)	
H210	0.6246	0.4773	0.0514	0.062*	
C211	0.5083 (7)	0.5471 (4)	0.0686 (3)	0.049 (2)	
H211	0.4406	0.5252	0.0568	0.058*	
C212	0.4972 (7)	0.6083 (3)	0.0889 (3)	0.0442 (19)	
H212	0.4226	0.6275	0.0905	0.053*	
C301	0.8282 (5)	0.9035 (3)	0.3285 (3)	0.0337 (15)	
C302	0.9207 (6)	0.9468 (4)	0.3219 (3)	0.0456 (18)	
H302	0.9134	0.9804	0.2962	0.055*	
C303	1.0229 (7)	0.9394 (4)	0.3539 (4)	0.062 (2)	
H303	1.0854	0.9678	0.3491	0.075*	
C304	1.0344 (7)	0.8914 (4)	0.3922 (4)	0.061 (2)	
H304	1.1037	0.8875	0.4140	0.073*	
C305	0.9445 (8)	0.8489 (4)	0.3987 (4)	0.059 (2)	
H305	0.9527	0.8158	0.4249	0.071*	
C306	0.8417 (6)	0.8545 (4)	0.3672 (3)	0.0468 (19)	
H306	0.7807	0.8251	0.3718	0.056*	
C307	0.7004 (5)	0.9834 (3)	0.2503 (3)	0.0327 (16)	
C308	0.7103 (7)	0.9889 (4)	0.1914 (3)	0.0488 (19)	
H308	0.7171	0.9528	0.1687	0.059*	
C309	0.7102 (8)	1.0472 (4)	0.1662 (4)	0.064 (2)	
H309	0.7165	1.0507	0.1263	0.077*	
C310	0.7008 (8)	1.1003 (4)	0.1991 (5)	0.063 (2)	
H310	0.6989	1.1400	0.1817	0.076*	
C311	0.6945 (6)	1.0954 (3)	0.2570 (5)	0.057 (2)	
H311	0.6906	1.1319	0.2793	0.068*	
C312	0.6937 (6)	1.0374 (3)	0.2835 (3)	0.0430 (18)	
H312	0.6886	1.0345	0.3235	0.052*	
C401	0.3526 (5)	0.8256 (3)	0.3068 (3)	0.0329 (13)	
C402	0.2647 (6)	0.8044 (3)	0.2700 (3)	0.0443 (17)	
H402	0.2664	0.8155	0.2312	0.053*	
C403	0.1744 (6)	0.7669 (4)	0.2910 (3)	0.0487 (19)	
H403	0.1155	0.7523	0.2660	0.058*	
C404	0.1704 (6)	0.7508 (3)	0.3474 (3)	0.0480 (19)	
H404	0.1089	0.7254	0.3612	0.058*	
C405	0.2564 (7)	0.7720 (4)	0.3839 (3)	0.052 (2)	
H405	0.2534	0.7611	0.4227	0.063*	
C406	0.3477 (6)	0.8092 (3)	0.3640 (3)	0.0431 (17)	
H406	0.4063	0.8234	0.3893	0.052*	
C407	0.4038 (5)	0.9329 (3)	0.2365 (3)	0.0333 (14)	
C408	0.4072 (6)	0.9360 (4)	0.1777 (3)	0.0450 (18)	
H408	0.4447	0.9042	0.1567	0.054*	
C409	0.3554 (7)	0.9862 (4)	0.1495 (4)	0.059 (2)	
H409	0.3587	0.9887	0.1094	0.070*	
C410	0.2991 (7)	1.0324 (4)	0.1804 (4)	0.059 (2)	
H410	0.2646	1.0665	0.1613	0.071*	
C411	0.2931 (6)	1.0289 (3)	0.2386 (4)	0.054 (2)	
H411	0.2534	1.0602	0.2593	0.064*	
C412	0.3453 (5)	0.9795 (3)	0.2671 (4)	0.0438 (16)	
H412	0.3414	0.9773	0.3071	0.053*	
S1	0.65359 (18)	0.89662 (10)	−0.04084 (8)	0.0511 (5)	
O11	0.7181 (6)	0.8541 (3)	−0.0056 (3)	0.0777 (18)	
O12	0.5375 (6)	0.8767 (4)	−0.0554 (2)	0.083 (2)	
O13	0.7191 (5)	0.9229 (4)	−0.0871 (2)	0.075 (2)	
C8	0.6286 (11)	0.9612 (5)	0.0071 (4)	0.078 (3)	
F11	0.5598 (10)	1.0042 (4)	−0.0147 (4)	0.173 (4)	
F12	0.7279 (8)	0.9880 (3)	0.0216 (4)	0.128 (3)	
F13	0.5761 (7)	0.9421 (4)	0.0550 (3)	0.129 (3)	
O16	0.8412 (7)	0.6317 (4)	0.5110 (4)	0.105 (3)*	
C9	1.0478 (12)	0.6303 (7)	0.4772 (6)	0.102 (4)*	
F16	1.1332 (8)	0.6358 (4)	0.4399 (3)	0.132 (2)*	
S2	0.9061 (7)	0.6398 (3)	0.4604 (2)	0.0676 (15)	0.6667
O14	0.8900 (14)	0.5840 (7)	0.4274 (6)	0.131 (5)*	0.6667
O15	0.9063 (13)	0.6945 (7)	0.4257 (6)	0.108 (5)*	0.6667
F14	1.0955 (14)	0.6839 (8)	0.5076 (6)	0.177 (5)*	0.6667
F15	1.0841 (11)	0.5830 (6)	0.5099 (6)	0.118 (4)*	0.6667
S2A	0.9146 (14)	0.6654 (6)	0.4747 (5)	0.078 (4)	0.3333
O14A	0.944 (2)	0.7199 (12)	0.5017 (10)	0.097 (7)*	0.3333
O15A	0.872 (2)	0.6692 (11)	0.4132 (10)	0.084 (6)*	0.3333
F14A	1.034 (2)	0.5621 (12)	0.4516 (11)	0.135 (8)*	0.3333
F15A	1.0798 (15)	0.6106 (9)	0.5301 (8)	0.074 (5)*	0.3333

### [Bis(diphenylphosphanyl)methane]({[(diphenylphosphanyl)methyl]diphenylphosphanylidene}(ethoxyoxoethanylidene)methanylidene-κ3P,C,O)hydridoiridium(III) bis(trifluoromethanesulfonate) (3) Atomic displacement parameters (Å^2^)

**Table:**

	*U*^11^	*U*^22^	*U*^33^	*U*^12^	*U*^13^	*U*^23^
Ir1	0.02452 (10)	0.02423 (12)	0.02655 (11)	−0.00201 (10)	0.00015 (10)	−0.00194 (11)
P1	0.0248 (8)	0.0284 (9)	0.0273 (9)	−0.0015 (6)	−0.0004 (6)	−0.0012 (7)
P2	0.0296 (8)	0.0282 (9)	0.0278 (9)	−0.0045 (7)	−0.0017 (7)	−0.0029 (7)
P3	0.0300 (8)	0.0269 (9)	0.0304 (9)	−0.0027 (7)	−0.0017 (6)	−0.0045 (7)
P4	0.0261 (7)	0.0303 (9)	0.0303 (9)	0.0002 (6)	0.0011 (7)	−0.0032 (7)
O1	0.032 (2)	0.026 (2)	0.029 (2)	−0.002 (2)	0.003 (2)	−0.0014 (17)
O2	0.067 (3)	0.036 (3)	0.031 (2)	−0.006 (2)	0.004 (2)	0.006 (2)
C1	0.023 (3)	0.034 (4)	0.033 (3)	0.003 (3)	−0.001 (3)	−0.003 (3)
C2	0.025 (3)	0.031 (4)	0.032 (4)	−0.003 (3)	0.000 (3)	−0.001 (3)
C3	0.032 (4)	0.037 (4)	0.031 (4)	0.006 (3)	0.004 (3)	−0.005 (3)
C4	0.032 (3)	0.024 (4)	0.038 (4)	−0.003 (3)	0.001 (2)	−0.004 (3)
C5	0.033 (3)	0.037 (4)	0.033 (4)	0.000 (3)	0.004 (3)	0.001 (3)
C6	0.095 (6)	0.055 (5)	0.028 (4)	−0.002 (5)	0.004 (4)	0.006 (4)
C7	0.186 (13)	0.085 (8)	0.038 (5)	−0.010 (8)	0.027 (7)	0.018 (5)
C101	0.025 (3)	0.034 (4)	0.036 (4)	−0.003 (2)	0.004 (3)	0.002 (3)
C102	0.033 (4)	0.050 (5)	0.045 (4)	−0.012 (3)	−0.006 (3)	0.002 (4)
C103	0.049 (5)	0.055 (5)	0.065 (6)	−0.021 (4)	−0.015 (4)	0.012 (5)
C104	0.050 (5)	0.061 (6)	0.082 (7)	−0.026 (4)	−0.013 (5)	0.025 (5)
C105	0.066 (6)	0.091 (8)	0.065 (6)	−0.039 (5)	−0.009 (5)	0.042 (6)
C106	0.044 (4)	0.075 (6)	0.048 (5)	−0.027 (4)	−0.012 (3)	0.019 (4)
C107	0.022 (3)	0.032 (3)	0.032 (3)	−0.003 (3)	−0.001 (3)	−0.002 (3)
C108	0.029 (3)	0.040 (3)	0.031 (3)	−0.002 (3)	0.005 (4)	−0.004 (3)
C109	0.030 (3)	0.047 (4)	0.037 (4)	−0.004 (3)	−0.004 (3)	0.012 (3)
C110	0.040 (4)	0.038 (4)	0.048 (5)	0.005 (3)	−0.007 (3)	0.006 (3)
C111	0.035 (4)	0.050 (5)	0.041 (4)	0.012 (3)	0.000 (3)	−0.010 (4)
C112	0.032 (3)	0.042 (4)	0.032 (3)	0.004 (3)	0.003 (3)	−0.005 (3)
C201	0.030 (3)	0.031 (4)	0.037 (4)	−0.004 (3)	−0.004 (3)	−0.005 (3)
C202	0.036 (4)	0.060 (5)	0.050 (4)	0.001 (4)	0.003 (4)	0.007 (4)
C203	0.034 (4)	0.072 (6)	0.065 (6)	0.007 (4)	−0.003 (4)	0.000 (5)
C204	0.041 (4)	0.067 (6)	0.065 (5)	0.006 (4)	−0.011 (4)	−0.003 (5)
C205	0.054 (4)	0.078 (6)	0.048 (4)	0.004 (6)	−0.017 (4)	0.020 (4)
C206	0.044 (4)	0.045 (4)	0.039 (4)	−0.002 (3)	−0.006 (3)	0.009 (4)
C207	0.045 (4)	0.026 (4)	0.029 (3)	−0.005 (3)	−0.007 (3)	0.005 (3)
C208	0.044 (4)	0.036 (5)	0.053 (5)	0.001 (3)	−0.012 (4)	−0.003 (4)
C209	0.060 (5)	0.040 (5)	0.058 (5)	0.009 (4)	−0.010 (4)	−0.002 (4)
C210	0.076 (6)	0.029 (4)	0.051 (4)	0.000 (4)	−0.009 (4)	−0.006 (3)
C211	0.058 (5)	0.035 (5)	0.052 (5)	−0.014 (4)	−0.009 (4)	0.001 (4)
C212	0.044 (4)	0.036 (5)	0.053 (5)	−0.007 (3)	0.000 (3)	−0.005 (4)
C301	0.035 (4)	0.031 (4)	0.034 (4)	−0.002 (3)	−0.006 (3)	−0.006 (3)
C302	0.045 (4)	0.040 (4)	0.052 (5)	−0.005 (3)	−0.011 (4)	−0.002 (4)
C303	0.042 (5)	0.069 (6)	0.076 (6)	−0.013 (4)	−0.020 (4)	0.000 (5)
C304	0.051 (5)	0.068 (6)	0.064 (6)	0.008 (4)	−0.028 (4)	−0.005 (5)
C305	0.069 (6)	0.049 (5)	0.059 (5)	0.008 (4)	−0.026 (4)	0.002 (4)
C306	0.047 (5)	0.050 (5)	0.044 (4)	−0.006 (4)	−0.012 (3)	0.000 (4)
C307	0.027 (3)	0.031 (4)	0.040 (4)	−0.004 (3)	−0.002 (3)	−0.002 (3)
C308	0.065 (5)	0.034 (4)	0.047 (5)	−0.013 (4)	−0.002 (4)	0.000 (4)
C309	0.086 (6)	0.048 (5)	0.059 (5)	−0.020 (5)	−0.017 (5)	0.018 (5)
C310	0.060 (5)	0.043 (5)	0.088 (7)	−0.009 (4)	−0.022 (5)	0.021 (5)
C311	0.041 (4)	0.028 (4)	0.102 (8)	0.002 (3)	−0.015 (5)	−0.013 (5)
C312	0.040 (4)	0.037 (4)	0.053 (5)	−0.004 (3)	−0.005 (3)	−0.010 (4)
C401	0.032 (3)	0.030 (3)	0.037 (3)	0.001 (3)	0.004 (3)	−0.002 (3)
C402	0.043 (4)	0.049 (5)	0.040 (4)	−0.010 (3)	−0.004 (3)	0.010 (4)
C403	0.039 (4)	0.046 (4)	0.061 (5)	−0.013 (3)	−0.006 (3)	0.002 (4)
C404	0.043 (4)	0.041 (4)	0.060 (5)	−0.006 (3)	0.008 (4)	0.009 (4)
C405	0.059 (5)	0.058 (5)	0.041 (5)	−0.017 (4)	0.010 (4)	0.011 (4)
C406	0.044 (4)	0.049 (5)	0.037 (4)	−0.006 (4)	0.003 (3)	−0.003 (3)
C407	0.027 (3)	0.030 (3)	0.042 (4)	−0.004 (2)	−0.003 (3)	−0.001 (3)
C408	0.040 (4)	0.050 (5)	0.045 (4)	0.004 (3)	0.002 (3)	0.001 (4)
C409	0.047 (4)	0.067 (6)	0.063 (5)	0.006 (4)	0.004 (4)	0.027 (4)
C410	0.045 (4)	0.040 (5)	0.091 (7)	−0.004 (4)	−0.012 (5)	0.016 (5)
C411	0.039 (4)	0.042 (4)	0.080 (6)	0.008 (3)	−0.014 (4)	−0.016 (5)
C412	0.042 (4)	0.033 (4)	0.056 (4)	0.005 (3)	0.001 (4)	−0.004 (4)
S1	0.0523 (12)	0.0681 (13)	0.0328 (9)	−0.0041 (10)	−0.0028 (9)	0.0017 (9)
O11	0.102 (5)	0.072 (4)	0.058 (4)	0.014 (4)	−0.020 (4)	0.005 (3)
O12	0.069 (4)	0.141 (7)	0.040 (3)	−0.040 (4)	−0.002 (3)	−0.005 (4)
O13	0.059 (4)	0.123 (6)	0.043 (3)	−0.008 (4)	0.011 (3)	0.015 (4)
C8	0.087 (7)	0.081 (7)	0.066 (6)	0.010 (7)	−0.016 (6)	−0.006 (5)
F11	0.229 (10)	0.135 (7)	0.156 (8)	0.112 (7)	−0.017 (7)	−0.016 (6)
F12	0.152 (7)	0.099 (6)	0.132 (6)	−0.041 (5)	−0.004 (5)	−0.044 (5)
F13	0.144 (6)	0.174 (8)	0.068 (4)	−0.017 (5)	0.042 (4)	−0.034 (5)
S2	0.112 (4)	0.056 (3)	0.034 (3)	0.005 (3)	0.003 (3)	0.004 (2)
S2A	0.114 (7)	0.086 (10)	0.034 (5)	−0.020 (8)	0.009 (5)	−0.008 (5)

### [Bis(diphenylphosphanyl)methane]({[(diphenylphosphanyl)methyl]diphenylphosphanylidene}(ethoxyoxoethanylidene)methanylidene-κ3P,C,O)hydridoiridium(III) bis(trifluoromethanesulfonate) (3) Geometric parameters (Å, º)

**Table:**

Ir1—C1	2.062 (6)	C208—H208	0.9400
Ir1—O1	2.239 (4)	C209—C210	1.365 (11)
Ir1—P1	2.2945 (16)	C209—H209	0.9400
Ir1—P3	2.3330 (16)	C210—C211	1.384 (11)
Ir1—P4	2.3410 (15)	C210—H210	0.9400
Ir1—H1	1.58 (5)	C211—C212	1.396 (11)
P1—C101	1.809 (6)	C211—H211	0.9400
P1—C107	1.812 (6)	C212—H212	0.9400
P1—C2	1.852 (6)	C301—C306	1.393 (10)
P2—C1	1.789 (7)	C301—C302	1.406 (9)
P2—C201	1.794 (7)	C302—C303	1.387 (11)
P2—C207	1.797 (7)	C302—H302	0.9400
P2—C2	1.824 (6)	C303—C304	1.369 (12)
P3—C307	1.801 (7)	C303—H303	0.9400
P3—C301	1.807 (6)	C304—C305	1.372 (12)
P3—C3	1.838 (6)	C304—H304	0.9400
P3—P4	2.688 (2)	C305—C306	1.384 (10)
P4—C401	1.806 (6)	C305—H305	0.9400
P4—C407	1.813 (7)	C306—H306	0.9400
P4—C3	1.837 (7)	C307—C308	1.389 (10)
O1—C5	1.248 (8)	C307—C312	1.393 (9)
O2—C5	1.302 (8)	C308—C309	1.379 (11)
O2—C6	1.464 (8)	C308—H308	0.9400
C1—C4	1.335 (9)	C309—C310	1.376 (13)
C2—H2A	0.9800	C309—H309	0.9400
C2—H2B	0.9800	C310—C311	1.360 (12)
C3—H3A	0.9800	C310—H310	0.9400
C3—H3B	0.9800	C311—C312	1.386 (11)
C4—C5	1.460 (9)	C311—H311	0.9400
C4—H4	0.95 (7)	C312—H312	0.9400
C6—C7	1.492 (12)	C401—C406	1.385 (9)
C6—H6A	0.9800	C401—C402	1.391 (9)
C6—H6B	0.9800	C402—C403	1.389 (9)
C7—H7A	0.9700	C402—H402	0.9400
C7—H7B	0.9700	C403—C404	1.365 (11)
C7—H7C	0.9700	C403—H403	0.9400
C101—C102	1.376 (9)	C404—C405	1.373 (11)
C101—C106	1.381 (9)	C404—H404	0.9400
C102—C103	1.396 (10)	C405—C406	1.385 (10)
C102—H102	0.9400	C405—H405	0.9400
C103—C104	1.368 (12)	C406—H406	0.9400
C103—H103	0.9400	C407—C408	1.380 (10)
C104—C105	1.343 (12)	C407—C412	1.393 (9)
C104—H104	0.9400	C408—C409	1.389 (10)
C105—C106	1.388 (11)	C408—H408	0.9400
C105—H105	0.9400	C409—C410	1.380 (12)
C106—H106	0.9400	C409—H409	0.9400
C107—C108	1.393 (8)	C410—C411	1.367 (12)
C107—C112	1.397 (9)	C410—H410	0.9400
C108—C109	1.388 (9)	C411—C412	1.382 (10)
C108—H108	0.9400	C411—H411	0.9400
C109—C110	1.373 (10)	C412—H412	0.9400
C109—H109	0.9400	S1—O12	1.423 (6)
C110—C111	1.370 (10)	S1—O13	1.427 (6)
C110—H110	0.9400	S1—O11	1.429 (6)
C111—C112	1.386 (10)	S1—C8	1.802 (10)
C111—H111	0.9400	C8—F12	1.306 (13)
C112—H112	0.9400	C8—F11	1.308 (12)
C201—C202	1.388 (9)	C8—F13	1.332 (12)
C201—C206	1.390 (10)	O16—S2A	1.389 (16)
C202—C203	1.391 (11)	O16—S2	1.406 (10)
C202—H202	0.9400	C9—F16	1.308 (14)
C203—C204	1.356 (12)	C9—F15	1.333 (16)
C203—H203	0.9400	C9—F15A	1.36 (2)
C204—C205	1.361 (11)	C9—F14	1.452 (19)
C204—H204	0.9400	C9—F14A	1.58 (3)
C205—C206	1.394 (10)	C9—S2	1.665 (16)
C205—H205	0.9400	C9—S2A	1.69 (2)
C206—H206	0.9400	S2—O15	1.424 (15)
C207—C212	1.385 (9)	S2—O14	1.433 (15)
C207—C208	1.398 (10)	S2A—O14A	1.37 (3)
C208—C209	1.384 (10)	S2A—O15A	1.52 (3)
			
C1—Ir1—O1	75.5 (2)	C204—C205—H205	119.5
C1—Ir1—P1	79.45 (17)	C206—C205—H205	119.5
O1—Ir1—P1	109.19 (11)	C201—C206—C205	118.1 (7)
C1—Ir1—P3	167.72 (19)	C201—C206—H206	120.9
O1—Ir1—P3	92.25 (11)	C205—C206—H206	120.9
P1—Ir1—P3	106.08 (6)	C212—C207—C208	119.3 (6)
C1—Ir1—P4	106.41 (17)	C212—C207—P2	120.2 (5)
O1—Ir1—P4	82.13 (11)	C208—C207—P2	120.5 (5)
P1—Ir1—P4	168.44 (6)	C209—C208—C207	119.7 (7)
P3—Ir1—P4	70.23 (6)	C209—C208—H208	120.2
C1—Ir1—H1	100.0 (18)	C207—C208—H208	120.2
O1—Ir1—H1	167.5 (17)	C210—C209—C208	121.6 (8)
P1—Ir1—H1	81.1 (17)	C210—C209—H209	119.2
P3—Ir1—H1	91.7 (17)	C208—C209—H209	119.2
P4—Ir1—H1	88.0 (17)	C209—C210—C211	118.8 (7)
C101—P1—C107	105.7 (3)	C209—C210—H210	120.6
C101—P1—C2	104.6 (3)	C211—C210—H210	120.6
C107—P1—C2	106.5 (3)	C210—C211—C212	120.9 (7)
C101—P1—Ir1	118.7 (2)	C210—C211—H211	119.5
C107—P1—Ir1	117.8 (2)	C212—C211—H211	119.5
C2—P1—Ir1	102.0 (2)	C207—C212—C211	119.6 (7)
C1—P2—C201	106.3 (3)	C207—C212—H212	120.2
C1—P2—C207	117.6 (3)	C211—C212—H212	120.2
C201—P2—C207	109.9 (3)	C306—C301—C302	119.0 (6)
C1—P2—C2	101.2 (3)	C306—C301—P3	120.0 (5)
C201—P2—C2	113.2 (3)	C302—C301—P3	120.8 (5)
C207—P2—C2	108.5 (3)	C303—C302—C301	119.1 (7)
C307—P3—C301	106.5 (3)	C303—C302—H302	120.4
C307—P3—C3	105.6 (3)	C301—C302—H302	120.4
C301—P3—C3	109.0 (3)	C304—C303—C302	121.2 (8)
C307—P3—Ir1	120.6 (2)	C304—C303—H303	119.4
C301—P3—Ir1	120.2 (2)	C302—C303—H303	119.4
C3—P3—Ir1	92.3 (2)	C303—C304—C305	119.9 (7)
C307—P3—P4	104.7 (2)	C303—C304—H304	120.1
C301—P3—P4	143.3 (2)	C305—C304—H304	120.1
C3—P3—P4	43.0 (2)	C304—C305—C306	120.5 (8)
Ir1—P3—P4	55.03 (5)	C304—C305—H305	119.7
C401—P4—C407	105.5 (3)	C306—C305—H305	119.7
C401—P4—C3	112.5 (3)	C305—C306—C301	120.2 (7)
C407—P4—C3	105.4 (3)	C305—C306—H306	119.9
C401—P4—Ir1	121.8 (2)	C301—C306—H306	119.9
C407—P4—Ir1	117.8 (2)	C308—C307—C312	119.2 (7)
C3—P4—Ir1	92.1 (2)	C308—C307—P3	120.7 (5)
C401—P4—P3	147.5 (2)	C312—C307—P3	120.1 (5)
C407—P4—P3	102.61 (19)	C309—C308—C307	120.1 (8)
C3—P4—P3	43.0 (2)	C309—C308—H308	119.9
Ir1—P4—P3	54.75 (5)	C307—C308—H308	119.9
C5—O1—Ir1	111.9 (4)	C310—C309—C308	120.3 (8)
C5—O2—C6	117.6 (6)	C310—C309—H309	119.8
C4—C1—P2	124.8 (5)	C308—C309—H309	119.8
C4—C1—Ir1	118.0 (5)	C311—C310—C309	119.9 (8)
P2—C1—Ir1	115.5 (3)	C311—C310—H310	120.0
P2—C2—P1	109.2 (3)	C309—C310—H310	120.0
P2—C2—H2A	109.8	C310—C311—C312	121.0 (8)
P1—C2—H2A	109.8	C310—C311—H311	119.5
P2—C2—H2B	109.8	C312—C311—H311	119.5
P1—C2—H2B	109.8	C311—C312—C307	119.4 (7)
H2A—C2—H2B	108.3	C311—C312—H312	120.3
P4—C3—P3	94.0 (3)	C307—C312—H312	120.3
P4—C3—H3A	112.9	C406—C401—C402	119.2 (6)
P3—C3—H3A	112.9	C406—C401—P4	123.5 (5)
P4—C3—H3B	112.9	C402—C401—P4	117.2 (5)
P3—C3—H3B	112.9	C403—C402—C401	119.7 (7)
H3A—C3—H3B	110.3	C403—C402—H402	120.1
C1—C4—C5	114.1 (6)	C401—C402—H402	120.1
C1—C4—H4	127 (4)	C404—C403—C402	120.7 (7)
C5—C4—H4	118 (4)	C404—C403—H403	119.6
O1—C5—O2	122.7 (6)	C402—C403—H403	119.6
O1—C5—C4	119.9 (6)	C403—C404—C405	119.7 (7)
O2—C5—C4	117.4 (6)	C403—C404—H404	120.2
O2—C6—C7	107.4 (7)	C405—C404—H404	120.2
O2—C6—H6A	110.2	C404—C405—C406	120.7 (7)
C7—C6—H6A	110.2	C404—C405—H405	119.7
O2—C6—H6B	110.2	C406—C405—H405	119.7
C7—C6—H6B	110.2	C401—C406—C405	119.9 (7)
H6A—C6—H6B	108.5	C401—C406—H406	120.0
C6—C7—H7A	109.5	C405—C406—H406	120.0
C6—C7—H7B	109.5	C408—C407—C412	119.5 (6)
H7A—C7—H7B	109.5	C408—C407—P4	121.8 (5)
C6—C7—H7C	109.5	C412—C407—P4	118.8 (6)
H7A—C7—H7C	109.5	C407—C408—C409	120.0 (7)
H7B—C7—H7C	109.5	C407—C408—H408	120.0
C102—C101—C106	118.9 (6)	C409—C408—H408	120.0
C102—C101—P1	117.5 (5)	C410—C409—C408	119.8 (8)
C106—C101—P1	123.4 (5)	C410—C409—H409	120.1
C101—C102—C103	120.5 (7)	C408—C409—H409	120.1
C101—C102—H102	119.8	C411—C410—C409	120.5 (8)
C103—C102—H102	119.8	C411—C410—H410	119.7
C104—C103—C102	119.4 (7)	C409—C410—H410	119.7
C104—C103—H103	120.3	C410—C411—C412	120.1 (8)
C102—C103—H103	120.3	C410—C411—H411	120.0
C105—C104—C103	120.4 (8)	C412—C411—H411	120.0
C105—C104—H104	119.8	C411—C412—C407	120.1 (8)
C103—C104—H104	119.8	C411—C412—H412	119.9
C104—C105—C106	121.1 (8)	C407—C412—H412	119.9
C104—C105—H105	119.4	O12—S1—O13	114.5 (4)
C106—C105—H105	119.4	O12—S1—O11	114.9 (5)
C101—C106—C105	119.6 (7)	O13—S1—O11	115.0 (4)
C101—C106—H106	120.2	O12—S1—C8	103.5 (5)
C105—C106—H106	120.2	O13—S1—C8	104.7 (5)
C108—C107—C112	118.4 (6)	O11—S1—C8	102.0 (4)
C108—C107—P1	119.2 (5)	F12—C8—F11	107.9 (10)
C112—C107—P1	122.3 (5)	F12—C8—F13	107.4 (9)
C109—C108—C107	120.5 (6)	F11—C8—F13	106.1 (10)
C109—C108—H108	119.8	F12—C8—S1	111.2 (8)
C107—C108—H108	119.8	F11—C8—S1	112.8 (8)
C110—C109—C108	119.9 (6)	F13—C8—S1	111.1 (8)
C110—C109—H109	120.1	F16—C9—F15	102.9 (12)
C108—C109—H109	120.1	F16—C9—F15A	116.0 (13)
C111—C110—C109	120.7 (7)	F16—C9—F14	89.0 (11)
C111—C110—H110	119.7	F15—C9—F14	101.6 (12)
C109—C110—H110	119.7	F16—C9—F14A	84.3 (13)
C110—C111—C112	120.0 (6)	F15A—C9—F14A	94.9 (15)
C110—C111—H111	120.0	F16—C9—S2	123.0 (11)
C112—C111—H111	120.0	F15—C9—S2	121.7 (11)
C111—C112—C107	120.5 (6)	F14—C9—S2	112.2 (11)
C111—C112—H112	119.8	F16—C9—S2A	126.9 (11)
C107—C112—H112	119.8	F15A—C9—S2A	114.1 (13)
C202—C201—C206	120.3 (6)	F14A—C9—S2A	108.1 (13)
C202—C201—P2	117.9 (5)	O16—S2—O15	125.6 (8)
C206—C201—P2	121.7 (5)	O16—S2—O14	106.6 (8)
C201—C202—C203	119.9 (7)	O15—S2—O14	112.1 (9)
C201—C202—H202	120.1	O16—S2—C9	106.9 (7)
C203—C202—H202	120.1	O15—S2—C9	103.5 (9)
C204—C203—C202	119.5 (8)	O14—S2—C9	98.6 (9)
C204—C203—H203	120.3	O14A—S2A—O16	107.7 (13)
C202—C203—H203	120.3	O14A—S2A—O15A	118.0 (16)
C203—C204—C205	121.3 (7)	O16—S2A—O15A	114.5 (14)
C203—C204—H204	119.4	O14A—S2A—C9	98.4 (14)
C205—C204—H204	119.4	O16—S2A—C9	106.6 (11)
C204—C205—C206	120.9 (7)	O15A—S2A—C9	110.0 (12)
			
C201—P2—C1—C4	113.3 (6)	C307—P3—C301—C302	−15.0 (7)
C207—P2—C1—C4	−10.3 (7)	C3—P3—C301—C302	−128.6 (6)
C2—P2—C1—C4	−128.3 (6)	Ir1—P3—C301—C302	126.9 (5)
C201—P2—C1—Ir1	−82.3 (4)	P4—P3—C301—C302	−162.2 (4)
C207—P2—C1—Ir1	154.1 (3)	C306—C301—C302—C303	0.3 (11)
C2—P2—C1—Ir1	36.1 (4)	P3—C301—C302—C303	−175.1 (6)
C1—P2—C2—P1	6.6 (4)	C301—C302—C303—C304	−1.1 (13)
C201—P2—C2—P1	119.9 (4)	C302—C303—C304—C305	1.1 (14)
C207—P2—C2—P1	−117.8 (3)	C303—C304—C305—C306	−0.4 (14)
C101—P1—C2—P2	−163.6 (3)	C304—C305—C306—C301	−0.3 (13)
C107—P1—C2—P2	84.7 (4)	C302—C301—C306—C305	0.4 (11)
Ir1—P1—C2—P2	−39.4 (3)	P3—C301—C306—C305	175.8 (6)
C401—P4—C3—P3	153.4 (3)	C301—P3—C307—C308	116.4 (6)
C407—P4—C3—P3	−92.1 (3)	C3—P3—C307—C308	−127.7 (6)
Ir1—P4—C3—P3	27.6 (3)	Ir1—P3—C307—C308	−25.3 (7)
C307—P3—C3—P4	95.0 (3)	P4—P3—C307—C308	−83.1 (6)
C301—P3—C3—P4	−150.9 (3)	C301—P3—C307—C312	−62.7 (6)
Ir1—P3—C3—P4	−27.7 (3)	C3—P3—C307—C312	53.2 (6)
P2—C1—C4—C5	167.5 (5)	Ir1—P3—C307—C312	155.5 (4)
Ir1—C1—C4—C5	3.5 (7)	P4—P3—C307—C312	97.7 (5)
Ir1—O1—C5—O2	−172.2 (5)	C312—C307—C308—C309	−1.8 (12)
Ir1—O1—C5—C4	9.3 (7)	P3—C307—C308—C309	179.1 (6)
C6—O2—C5—O1	−1.7 (10)	C307—C308—C309—C310	0.3 (13)
C6—O2—C5—C4	176.9 (6)	C308—C309—C310—C311	1.6 (14)
C1—C4—C5—O1	−9.0 (9)	C309—C310—C311—C312	−2.0 (13)
C1—C4—C5—O2	172.5 (6)	C310—C311—C312—C307	0.5 (11)
C5—O2—C6—C7	176.9 (8)	C308—C307—C312—C311	1.4 (10)
C107—P1—C101—C102	−56.6 (6)	P3—C307—C312—C311	−179.5 (5)
C2—P1—C101—C102	−168.8 (5)	C407—P4—C401—C406	−129.5 (6)
Ir1—P1—C101—C102	78.4 (6)	C3—P4—C401—C406	−15.1 (7)
C107—P1—C101—C106	127.3 (6)	Ir1—P4—C401—C406	92.6 (6)
C2—P1—C101—C106	15.1 (7)	P3—P4—C401—C406	19.6 (8)
Ir1—P1—C101—C106	−97.7 (6)	C407—P4—C401—C402	54.4 (6)
C106—C101—C102—C103	2.3 (11)	C3—P4—C401—C402	168.9 (5)
P1—C101—C102—C103	−174.1 (6)	Ir1—P4—C401—C402	−83.4 (5)
C101—C102—C103—C104	1.1 (13)	P3—P4—C401—C402	−156.5 (4)
C102—C103—C104—C105	−3.6 (15)	C406—C401—C402—C403	−0.7 (10)
C103—C104—C105—C106	2.8 (17)	P4—C401—C402—C403	175.6 (6)
C102—C101—C106—C105	−3.1 (12)	C401—C402—C403—C404	0.5 (11)
P1—C101—C106—C105	173.0 (7)	C402—C403—C404—C405	−0.1 (12)
C104—C105—C106—C101	0.6 (15)	C403—C404—C405—C406	−0.3 (12)
C101—P1—C107—C108	116.2 (5)	C402—C401—C406—C405	0.3 (11)
C2—P1—C107—C108	−132.9 (5)	P4—C401—C406—C405	−175.6 (6)
Ir1—P1—C107—C108	−19.2 (6)	C404—C405—C406—C401	0.2 (12)
C101—P1—C107—C112	−63.9 (6)	C401—P4—C407—C408	−112.2 (6)
C2—P1—C107—C112	47.0 (6)	C3—P4—C407—C408	128.5 (5)
Ir1—P1—C107—C112	160.7 (4)	Ir1—P4—C407—C408	27.6 (6)
C112—C107—C108—C109	−1.9 (9)	P3—P4—C407—C408	84.2 (5)
P1—C107—C108—C109	178.0 (5)	C401—P4—C407—C412	67.4 (5)
C107—C108—C109—C110	−0.7 (10)	C3—P4—C407—C412	−51.9 (5)
C108—C109—C110—C111	2.5 (10)	Ir1—P4—C407—C412	−152.8 (4)
C109—C110—C111—C112	−1.7 (11)	P3—P4—C407—C412	−96.2 (5)
C110—C111—C112—C107	−1.0 (10)	C412—C407—C408—C409	1.7 (10)
C108—C107—C112—C111	2.7 (9)	P4—C407—C408—C409	−178.7 (6)
P1—C107—C112—C111	−177.2 (5)	C407—C408—C409—C410	−0.9 (12)
C1—P2—C201—C202	−40.4 (6)	C408—C409—C410—C411	−0.5 (12)
C207—P2—C201—C202	87.8 (6)	C409—C410—C411—C412	1.1 (12)
C2—P2—C201—C202	−150.7 (5)	C410—C411—C412—C407	−0.3 (10)
C1—P2—C201—C206	140.5 (6)	C408—C407—C412—C411	−1.2 (9)
C207—P2—C201—C206	−91.2 (6)	P4—C407—C412—C411	179.2 (5)
C2—P2—C201—C206	30.3 (7)	O12—S1—C8—F12	−175.5 (8)
C206—C201—C202—C203	−0.4 (11)	O13—S1—C8—F12	−55.3 (9)
P2—C201—C202—C203	−179.4 (6)	O11—S1—C8—F12	64.9 (9)
C201—C202—C203—C204	−1.4 (13)	O12—S1—C8—F11	−54.1 (10)
C202—C203—C204—C205	1.6 (14)	O13—S1—C8—F11	66.1 (10)
C203—C204—C205—C206	0.0 (14)	O11—S1—C8—F11	−173.7 (9)
C202—C201—C206—C205	1.9 (11)	O12—S1—C8—F13	64.9 (8)
P2—C201—C206—C205	−179.1 (6)	O13—S1—C8—F13	−174.9 (7)
C204—C205—C206—C201	−1.8 (13)	O11—S1—C8—F13	−54.7 (9)
C1—P2—C207—C212	97.3 (6)	F16—C9—S2—O16	−178.8 (11)
C201—P2—C207—C212	−24.4 (7)	F15—C9—S2—O16	45.8 (15)
C2—P2—C207—C212	−148.7 (6)	F14—C9—S2—O16	−74.8 (12)
C1—P2—C207—C208	−83.8 (6)	F16—C9—S2—O15	−44.5 (15)
C201—P2—C207—C208	154.5 (6)	F15—C9—S2—O15	−179.9 (13)
C2—P2—C207—C208	30.2 (7)	F14—C9—S2—O15	59.5 (13)
C212—C207—C208—C209	2.1 (11)	F16—C9—S2—O14	70.8 (14)
P2—C207—C208—C209	−176.8 (6)	F15—C9—S2—O14	−64.6 (15)
C207—C208—C209—C210	−1.7 (12)	F14—C9—S2—O14	174.9 (12)
C208—C209—C210—C211	0.3 (12)	F16—C9—S2A—O14A	−87.5 (17)
C209—C210—C211—C212	0.9 (12)	F15A—C9—S2A—O14A	71.8 (18)
C208—C207—C212—C211	−1.0 (11)	F14A—C9—S2A—O14A	175.9 (16)
P2—C207—C212—C211	177.9 (6)	F16—C9—S2A—O16	161.1 (12)
C210—C211—C212—C207	−0.5 (12)	F15A—C9—S2A—O16	−39.5 (16)
C307—P3—C301—C306	169.7 (6)	F14A—C9—S2A—O16	64.6 (14)
C3—P3—C301—C306	56.2 (6)	F16—C9—S2A—O15A	36 (2)
Ir1—P3—C301—C306	−48.4 (6)	F15A—C9—S2A—O15A	−164.3 (16)
P4—P3—C301—C306	22.5 (8)	F14A—C9—S2A—O15A	−60.1 (18)

### [Bis(diphenylphosphanyl)methane]({[(diphenylphosphanyl)methyl]diphenylphosphanylidene}(ethoxyoxoethanylidene)methanylidene-κ3P,C,O)hydridoiridium(III) bis(trifluoromethanesulfonate) (3) Hydrogen-bond geometry (Å, º)

**Table:**

*D*—H···*A*	*D*—H	H···*A*	*D*···*A*	*D*—H···*A*
C2—H2*A*···O11	0.98	2.53	3.420 (9)	151
C3—H3*A*···F15*A*^i^	0.98	2.48	3.310 (19)	142
C6—H6*A*···O14*A*^i^	0.98	2.55	3.34 (3)	138
C6—H6*B*···O15*A*	0.98	2.38	3.32 (3)	160
C106—H106···O11	0.94	2.39	3.293 (10)	162
C108—H108···O1	0.94	2.43	3.255 (8)	147
C112—H112···O12^ii^	0.94	2.55	3.164 (9)	123
C206—H206···O11	0.94	2.61	3.473 (10)	152
C212—H212···O13^iii^	0.94	2.55	3.220 (9)	129
C306—H306···O1	0.94	2.61	3.396 (8)	141
C312—H312···O13^iv^	0.94	2.51	3.298 (9)	141
C406—H406···O16^i^	0.94	2.63	3.188 (11)	119

Symmetry codes: (i) *x*−1/2, −*y*+3/2, −*z*+1; (ii) *x*+1/2, −*y*+3/2, −*z*; (iii) *x*−1/2, −*y*+3/2, −*z*; (iv) −*x*+3/2, −*y*+2, *z*+1/2.

### [Bis(diphenylphosphanyl)methane]carbonyl({[(diphenylphosphanyl)methyl]diphenylphosphanylidene}(ethoxyoxoethanylidene)methanylidene- κ2P,C}hydridoiridium(III) bis(trifluoromethanesulfonate)–dichloromethane–ethyl acetate (6/2/3) (4) Crystal data

**Table:**

[IrH(C_30_H_28_O_2_P_2_)(C_25_H_22_P_2_)(CO)](CF_3_O_3_S)_2_·0.33CH_2_Cl_2_·0.5C_4_H_8_O_2_	*F*(000) = 5856
*M**_r_* = 1458.54	*D*_x_ = 1.569 Mg m^−^^3^
Monoclinic, *P*2_1_/*c*	Mo *K*α radiation, λ = 0.71073 Å
*a* = 14.4712 (3) Å	Cell parameters from 107278 reflections
*b* = 20.9611 (5) Å	θ = 1.0–26.0°
*c* = 41.3651 (9) Å	µ = 2.44 mm^−^^1^
β = 100.108 (1)°	*T* = 233 K
*V* = 12352.6 (5) Å^3^	Plate, colourless
*Z* = 8	0.15 × 0.09 × 0.04 mm

### [Bis(diphenylphosphanyl)methane]carbonyl({[(diphenylphosphanyl)methyl]diphenylphosphanylidene}(ethoxyoxoethanylidene)methanylidene- κ2P,C}hydridoiridium(III) bis(trifluoromethanesulfonate)–dichloromethane–ethyl acetate (6/2/3) (4) Data collection

**Table:**

Nonius KapppCCD diffractometer	*R*_int_ = 0.068
Detector resolution: 9.4 pixels mm^-1^	θ_max_ = 24.0°, θ_min_ = 1.1°
phi– and ω–scans	*h* = −16→16
45373 measured reflections	*k* = −23→23
18805 independent reflections	*l* = −47→47
14603 reflections with *I* > 2σ(*I*)	

### [Bis(diphenylphosphanyl)methane]carbonyl({[(diphenylphosphanyl)methyl]diphenylphosphanylidene}(ethoxyoxoethanylidene)methanylidene- κ2P,C}hydridoiridium(III) bis(trifluoromethanesulfonate)–dichloromethane–ethyl acetate (6/2/3) (4) Refinement

**Table:**

Refinement on *F*^2^	Primary atom site location: structure-invariant direct methods
Least-squares matrix: full	Hydrogen site location: mixed
*R*[*F*^2^ > 2σ(*F*^2^)] = 0.054	H atoms treated by a mixture of independent and constrained refinement
*wR*(*F*^2^) = 0.108	*w* = 1/[σ^2^(*F*_o_^2^) + (0.0273*P*)^2^ + 58.0653*P*] where *P* = (*F*_o_^2^ + 2*F*_c_^2^)/3
*S* = 1.04	(Δ/σ)_max_ = 0.006
18805 reflections	Δρ_max_ = 1.90 e Å^−^^3^
1530 parameters	Δρ_min_ = −0.93 e Å^−^^3^
5 restraints	

### [Bis(diphenylphosphanyl)methane]carbonyl({[(diphenylphosphanyl)methyl]diphenylphosphanylidene}(ethoxyoxoethanylidene)methanylidene- κ2P,C}hydridoiridium(III) bis(trifluoromethanesulfonate)–dichloromethane–ethyl acetate (6/2/3) (4) Special details

**Table:**

Geometry. All esds (except the esd in the dihedral angle between two l.s. planes) are estimated using the full covariance matrix. The cell esds are taken into account individually in the estimation of esds in distances, angles and torsion angles; correlations between esds in cell parameters are only used when they are defined by crystal symmetry. An approximate (isotropic) treatment of cell esds is used for estimating esds involving l.s. planes.
Refinement. Refined as a 2-component twin withPseudo merohedral twinning by matrix -1 0 0 0 -1 0 1 0 1. Small crystal with large lattice constant and two molecules into the asymmetric unit. Hydrid position at Ir-atoms were found and refined isotropically with bond restraint (d=1.6 angs.), whereas the temperature factor for H2 were fixed. The solvent molecule Dichloromethane was occupational disordered (occupation factor 0.5) and additional disordered in a second position nearby with occupation factor of around 0.166. This minor part were refined with bond restraints and isotropic displacement parameters. A second solvent ethyl acetate were ordered but refined with equal anisotropic displacement parameter for O21, C22 and C23.

### [Bis(diphenylphosphanyl)methane]carbonyl({[(diphenylphosphanyl)methyl]diphenylphosphanylidene}(ethoxyoxoethanylidene)methanylidene- κ2P,C}hydridoiridium(III) bis(trifluoromethanesulfonate)–dichloromethane–ethyl acetate (6/2/3) (4) Fractional atomic coordinates and isotropic or equivalent isotropic displacement parameters (Å^2^)

**Table:**

	*x*	*y*	*z*	*U*_iso_*/*U*_eq_	Occ. (<1)
Ir1	0.41814 (3)	0.69605 (3)	0.61409 (2)	0.02487 (11)	
H1	0.424 (9)	0.7719 (11)	0.611 (3)	0.05 (4)*	
Ir2	0.19337 (3)	1.19644 (3)	0.61298 (2)	0.02541 (11)	
H2	0.181 (8)	1.2703 (15)	0.620 (3)	0.050*	
P1	0.26929 (19)	0.71723 (14)	0.58395 (7)	0.0251 (7)	
P2	0.2403 (2)	0.72239 (15)	0.65226 (7)	0.0292 (7)	
P3	0.5121 (2)	0.72449 (14)	0.57458 (7)	0.0275 (7)	
P4	0.57638 (19)	0.71357 (14)	0.63974 (7)	0.0308 (7)	
P5	0.3115 (2)	1.21829 (14)	0.58254 (7)	0.0285 (7)	
P6	0.4071 (2)	1.22598 (15)	0.65065 (8)	0.0294 (7)	
P7	0.06036 (19)	1.22341 (14)	0.57366 (7)	0.0282 (7)	
P8	0.06114 (19)	1.21397 (14)	0.63876 (7)	0.0311 (7)	
C1	0.3549 (8)	0.6837 (5)	0.6565 (2)	0.027 (3)	
C2	0.1847 (7)	0.7016 (7)	0.6116 (2)	0.033 (3)	
H2A	0.1279	0.7272	0.6050	0.040*	
H2B	0.1669	0.6564	0.6106	0.040*	
C3	0.5987 (8)	0.7639 (5)	0.6056 (3)	0.034 (3)	
H3A	0.5840	0.8089	0.6085	0.040*	
H3B	0.6629	0.7596	0.6013	0.040*	
C4	0.3744 (7)	0.6472 (5)	0.6835 (3)	0.033 (3)	
H4	0.3306	0.6473	0.6977	0.039*	
C5	0.4576 (9)	0.6073 (6)	0.6927 (3)	0.046 (3)	
C6	0.5296 (10)	0.5339 (8)	0.7327 (4)	0.086 (6)	
H6A	0.5362	0.5039	0.7151	0.103*	
H6B	0.5889	0.5572	0.7386	0.103*	
C7	0.5128 (15)	0.5008 (11)	0.7593 (5)	0.167 (12)	
H7A	0.5641	0.4715	0.7664	0.251*	
H7B	0.5074	0.5303	0.7769	0.251*	
H7C	0.4547	0.4771	0.7535	0.251*	
C8	0.4137 (9)	0.6031 (7)	0.6080 (3)	0.034 (3)	
C11	0.3024 (7)	1.1861 (5)	0.6553 (2)	0.025 (3)	
C12	0.4207 (6)	1.2008 (7)	0.6108 (2)	0.030 (3)	
H12A	0.4339	1.1549	0.6108	0.036*	
H12B	0.4732	1.2235	0.6039	0.036*	
C13	0.0053 (8)	1.2637 (6)	0.6047 (3)	0.036 (3)	
H13A	0.0233	1.3087	0.6075	0.043*	
H13B	−0.0633	1.2597	0.6005	0.043*	
C14	0.3059 (8)	1.1541 (5)	0.6834 (3)	0.033 (3)	
H14	0.3595	1.1606	0.6995	0.039*	
C15	0.2340 (8)	1.1093 (6)	0.6920 (3)	0.038 (3)	
C16	0.1814 (11)	1.0559 (8)	0.7363 (3)	0.078 (5)	
H16A	0.1967	1.0114	0.7325	0.094*	
H16B	0.1173	1.0647	0.7251	0.094*	
C17	0.1910 (12)	1.0691 (9)	0.7725 (4)	0.105 (6)	
H17A	0.1483	1.0419	0.7818	0.157*	
H17B	0.2550	1.0604	0.7832	0.157*	
H17C	0.1761	1.1134	0.7758	0.157*	
C18	0.1982 (9)	1.1037 (6)	0.6071 (3)	0.028 (3)	
C101	0.2341 (7)	0.6690 (6)	0.5484 (3)	0.030 (3)	
C102	0.2161 (10)	0.6033 (6)	0.5513 (3)	0.041 (4)	
H102	0.2187	0.5847	0.5722	0.049*	
C103	0.1945 (8)	0.5667 (6)	0.5233 (3)	0.040 (3)	
H103	0.1829	0.5229	0.5253	0.048*	
C104	0.1893 (9)	0.5925 (7)	0.4922 (3)	0.050 (4)	
H104	0.1728	0.5669	0.4734	0.060*	
C105	0.2088 (10)	0.6566 (7)	0.4894 (3)	0.056 (4)	
H105	0.2076	0.6746	0.4685	0.067*	
C106	0.2300 (7)	0.6943 (6)	0.5170 (2)	0.035 (3)	
H106	0.2420	0.7379	0.5146	0.042*	
C107	0.2442 (8)	0.7988 (6)	0.5709 (2)	0.036 (3)	
C108	0.3039 (8)	0.8498 (6)	0.5796 (3)	0.034 (3)	
H108	0.3639	0.8421	0.5919	0.041*	
C109	0.2780 (9)	0.9118 (6)	0.5706 (3)	0.045 (3)	
H109	0.3201	0.9455	0.5771	0.053*	
C110	0.1895 (9)	0.9245 (6)	0.5520 (3)	0.050 (4)	
H110	0.1716	0.9662	0.5453	0.060*	
C111	0.1288 (10)	0.8727 (6)	0.5438 (3)	0.056 (4)	
H111	0.0686	0.8799	0.5316	0.068*	
C112	0.1550 (8)	0.8124 (6)	0.5531 (3)	0.042 (3)	
H112	0.1121	0.7789	0.5474	0.050*	
C201	0.1701 (7)	0.6953 (6)	0.6815 (3)	0.033 (3)	
C202	0.1347 (8)	0.6338 (6)	0.6801 (3)	0.043 (3)	
H202	0.1451	0.6057	0.6633	0.052*	
C203	0.0834 (9)	0.6141 (7)	0.7037 (3)	0.055 (4)	
H203	0.0583	0.5726	0.7027	0.066*	
C204	0.0693 (10)	0.6537 (7)	0.7281 (3)	0.057 (4)	
H204	0.0360	0.6384	0.7441	0.068*	
C205	0.1011 (10)	0.7147 (8)	0.7305 (4)	0.064 (4)	
H205	0.0884	0.7422	0.7472	0.077*	
C206	0.1549 (8)	0.7352 (7)	0.7065 (3)	0.043 (4)	
H206	0.1803	0.7766	0.7078	0.052*	
C207	0.2533 (7)	0.8065 (6)	0.6563 (2)	0.030 (3)	
C208	0.3406 (8)	0.8317 (6)	0.6707 (3)	0.040 (3)	
H208	0.3915	0.8044	0.6779	0.048*	
C209	0.3512 (10)	0.8980 (7)	0.6740 (3)	0.051 (4)	
H209	0.4081	0.9151	0.6850	0.062*	
C210	0.2806 (9)	0.9367 (6)	0.6618 (3)	0.050 (4)	
H210	0.2900	0.9811	0.6627	0.060*	
C211	0.1952 (9)	0.9132 (6)	0.6479 (3)	0.049 (3)	
H211	0.1454	0.9413	0.6404	0.059*	
C212	0.1817 (8)	0.8483 (5)	0.6449 (3)	0.040 (3)	
H212	0.1229	0.8324	0.6349	0.047*	
C301	0.4780 (8)	0.7750 (6)	0.5394 (3)	0.030 (3)	
C302	0.4431 (9)	0.7492 (7)	0.5096 (3)	0.045 (4)	
H302	0.4373	0.7046	0.5074	0.054*	
C303	0.4156 (10)	0.7879 (9)	0.4821 (3)	0.065 (5)	
H303	0.3875	0.7705	0.4618	0.078*	
C304	0.4309 (10)	0.8514 (8)	0.4857 (4)	0.066 (5)	
H304	0.4171	0.8773	0.4669	0.079*	
C305	0.4651 (10)	0.8798 (8)	0.5149 (4)	0.065 (4)	
H305	0.4732	0.9242	0.5165	0.078*	
C306	0.4877 (9)	0.8405 (6)	0.5426 (3)	0.041 (3)	
H306	0.5095	0.8587	0.5633	0.049*	
C307	0.5690 (8)	0.6575 (6)	0.5590 (3)	0.034 (3)	
C308	0.5187 (10)	0.6026 (6)	0.5480 (3)	0.045 (3)	
H308	0.4536	0.6015	0.5475	0.054*	
C309	0.5624 (12)	0.5505 (7)	0.5379 (3)	0.063 (5)	
H309	0.5287	0.5132	0.5312	0.076*	
C310	0.6581 (16)	0.5542 (8)	0.5380 (4)	0.084 (6)	
H310	0.6881	0.5188	0.5306	0.100*	
C311	0.7097 (15)	0.6056 (10)	0.5481 (4)	0.079 (6)	
H311	0.7749	0.6056	0.5486	0.095*	
C312	0.6661 (8)	0.6580 (7)	0.5577 (3)	0.047 (4)	
H312	0.7011	0.6953	0.5636	0.056*	
C401	0.5999 (8)	0.7581 (5)	0.6772 (3)	0.032 (3)	
C402	0.5873 (9)	0.8235 (6)	0.6772 (3)	0.044 (3)	
H402	0.5688	0.8456	0.6573	0.053*	
C403	0.6025 (10)	0.8568 (7)	0.7071 (3)	0.059 (4)	
H403	0.5941	0.9013	0.7072	0.070*	
C404	0.6291 (11)	0.8255 (8)	0.7359 (3)	0.066 (5)	
H404	0.6420	0.8483	0.7557	0.079*	
C405	0.6369 (12)	0.7626 (7)	0.7359 (3)	0.068 (5)	
H405	0.6483	0.7410	0.7562	0.081*	
C406	0.6289 (11)	0.7271 (6)	0.7070 (3)	0.045 (4)	
H406	0.6427	0.6832	0.7076	0.054*	
C407	0.6739 (9)	0.6556 (6)	0.6432 (3)	0.038 (3)	
C408	0.6619 (8)	0.5922 (6)	0.6365 (3)	0.041 (3)	
H408	0.6011	0.5751	0.6310	0.049*	
C409	0.7409 (11)	0.5521 (7)	0.6377 (3)	0.062 (4)	
H409	0.7345	0.5083	0.6329	0.075*	
C410	0.8288 (10)	0.5807 (10)	0.6464 (4)	0.071 (5)	
H410	0.8826	0.5549	0.6478	0.086*	
C411	0.8404 (10)	0.6448 (9)	0.6530 (4)	0.077 (6)	
H411	0.9016	0.6613	0.6585	0.092*	
C412	0.7628 (9)	0.6871 (9)	0.6519 (3)	0.062 (5)	
H412	0.7692	0.7310	0.6564	0.075*	
C501	0.3143 (8)	1.1688 (5)	0.5462 (3)	0.031 (3)	
C502	0.2864 (7)	1.1929 (7)	0.5151 (3)	0.041 (3)	
H502	0.2705	1.2363	0.5124	0.049*	
C503	0.2816 (9)	1.1539 (7)	0.4877 (3)	0.053 (4)	
H503	0.2635	1.1709	0.4665	0.064*	
C504	0.3031 (9)	1.0914 (7)	0.4916 (3)	0.055 (4)	
H504	0.2981	1.0648	0.4730	0.066*	
C505	0.3319 (10)	1.0661 (7)	0.5222 (3)	0.052 (4)	
H505	0.3481	1.0226	0.5245	0.062*	
C506	0.3375 (9)	1.1037 (6)	0.5497 (3)	0.031 (3)	
H506	0.3568	1.0861	0.5707	0.037*	
C507	0.3247 (7)	1.2982 (6)	0.5699 (3)	0.030 (3)	
C508	0.2717 (8)	1.3511 (6)	0.5779 (3)	0.038 (3)	
H508	0.2230	1.3442	0.5898	0.046*	
C509	0.2897 (9)	1.4119 (6)	0.5686 (3)	0.045 (3)	
H509	0.2545	1.4465	0.5744	0.054*	
C510	0.3603 (10)	1.4217 (7)	0.5506 (3)	0.054 (4)	
H510	0.3714	1.4633	0.5437	0.064*	
C511	0.4130 (9)	1.3744 (6)	0.5428 (3)	0.048 (3)	
H511	0.4620	1.3832	0.5313	0.057*	
C512	0.3966 (8)	1.3113 (6)	0.5515 (3)	0.042 (3)	
H512	0.4329	1.2778	0.5452	0.050*	
C601	0.5074 (7)	1.2037 (6)	0.6803 (2)	0.032 (3)	
C602	0.5442 (9)	1.1435 (6)	0.6780 (3)	0.043 (3)	
H602	0.5177	1.1162	0.6608	0.052*	
C603	0.6194 (8)	1.1233 (7)	0.7008 (3)	0.051 (4)	
H603	0.6438	1.0820	0.6995	0.061*	
C604	0.6579 (9)	1.1634 (8)	0.7251 (3)	0.055 (4)	
H604	0.7117	1.1510	0.7399	0.066*	
C605	0.6192 (11)	1.2217 (8)	0.7282 (4)	0.064 (5)	
H605	0.6421	1.2473	0.7465	0.077*	
C606	0.5485 (13)	1.2429 (7)	0.7052 (4)	0.063 (5)	
H606	0.5270	1.2850	0.7062	0.076*	
C607	0.3921 (8)	1.3107 (6)	0.6538 (3)	0.035 (3)	
C608	0.3220 (8)	1.3362 (6)	0.6693 (3)	0.038 (3)	
H608	0.2811	1.3090	0.6780	0.046*	
C609	0.3131 (9)	1.4007 (6)	0.6716 (3)	0.044 (3)	
H609	0.2634	1.4179	0.6807	0.053*	
C610	0.3761 (10)	1.4404 (6)	0.6608 (3)	0.051 (4)	
H610	0.3698	1.4848	0.6629	0.061*	
C611	0.4486 (10)	1.4163 (6)	0.6470 (3)	0.058 (4)	
H611	0.4922	1.4445	0.6403	0.070*	
C612	0.4586 (8)	1.3509 (6)	0.6428 (3)	0.042 (3)	
H612	0.5075	1.3342	0.6330	0.051*	
C701	0.0582 (7)	1.2756 (6)	0.5387 (3)	0.034 (3)	
C702	0.0651 (9)	1.2469 (7)	0.5075 (3)	0.049 (4)	
H702	0.0709	1.2025	0.5055	0.059*	
C703	0.0632 (10)	1.2858 (7)	0.4810 (3)	0.054 (4)	
H703	0.0652	1.2673	0.4604	0.064*	
C704	0.0583 (8)	1.3510 (8)	0.4832 (3)	0.054 (4)	
H704	0.0606	1.3769	0.4648	0.065*	
C705	0.0500 (9)	1.3783 (7)	0.5135 (3)	0.051 (4)	
H705	0.0451	1.4229	0.5152	0.061*	
C706	0.0488 (9)	1.3412 (7)	0.5405 (3)	0.047 (4)	
H706	0.0415	1.3603	0.5605	0.057*	
C707	−0.0119 (8)	1.1561 (5)	0.5581 (3)	0.030 (3)	
C708	0.0287 (9)	1.1018 (6)	0.5474 (3)	0.045 (3)	
H708	0.0935	1.1011	0.5471	0.053*	
C709	−0.0251 (11)	1.0490 (7)	0.5373 (3)	0.066 (4)	
H709	0.0041	1.0126	0.5305	0.079*	
C710	−0.1193 (11)	1.0477 (7)	0.5370 (4)	0.065 (5)	
H710	−0.1553	1.0110	0.5307	0.078*	
C711	−0.1601 (11)	1.1033 (8)	0.5465 (4)	0.064 (6)	
H711	−0.2255	1.1042	0.5456	0.076*	
C712	−0.1089 (9)	1.1570 (7)	0.5572 (3)	0.054 (4)	
H712	−0.1385	1.1934	0.5638	0.065*	
C801	0.0780 (8)	1.2591 (6)	0.6769 (3)	0.041 (3)	
C802	0.0899 (9)	1.2271 (7)	0.7067 (3)	0.047 (4)	
H802	0.0882	1.1823	0.7066	0.057*	
C803	0.1036 (10)	1.2574 (8)	0.7355 (3)	0.058 (4)	
H803	0.1077	1.2346	0.7553	0.070*	
C804	0.1118 (10)	1.3230 (8)	0.7358 (4)	0.064 (5)	
H804	0.1278	1.3445	0.7559	0.076*	
C805	0.0965 (10)	1.3584 (7)	0.7061 (3)	0.060 (4)	
H805	0.0978	1.4033	0.7063	0.072*	
C806	0.0797 (9)	1.3256 (7)	0.6772 (3)	0.049 (4)	
H806	0.0692	1.3481	0.6572	0.059*	
C807	−0.0272 (8)	1.1554 (6)	0.6429 (3)	0.038 (3)	
C808	−0.0213 (9)	1.0926 (7)	0.6357 (3)	0.054 (4)	
H808	0.0335	1.0764	0.6294	0.065*	
C809	−0.0964 (13)	1.0519 (7)	0.6377 (4)	0.070 (5)	
H809	−0.0910	1.0080	0.6338	0.084*	
C810	−0.1760 (12)	1.0754 (10)	0.6452 (4)	0.087 (6)	
H810	−0.2276	1.0480	0.6448	0.104*	
C811	−0.1849 (10)	1.1407 (10)	0.6537 (4)	0.078 (5)	
H811	−0.2399	1.1569	0.6598	0.093*	
C812	−0.1109 (9)	1.1773 (7)	0.6526 (3)	0.055 (4)	
H812	−0.1141	1.2204	0.6585	0.066*	
F1	−0.3899 (7)	1.0757 (5)	0.5979 (2)	0.093 (3)	
F2	−0.3351 (7)	0.9824 (5)	0.5937 (3)	0.101 (3)	
F3	−0.4628 (7)	1.0094 (4)	0.5635 (2)	0.083 (3)	
F4	−0.3549 (10)	1.3774 (6)	0.5590 (4)	0.139 (5)	
F5	−0.2357 (10)	1.4083 (6)	0.5923 (5)	0.204 (9)	
F6	−0.3768 (10)	1.4093 (5)	0.6060 (4)	0.135 (5)	
F7	0.0250 (6)	0.5087 (4)	0.5616 (2)	0.077 (3)	
F8	−0.0124 (7)	0.5743 (4)	0.5967 (2)	0.087 (3)	
F9	−0.0744 (6)	0.4813 (5)	0.5919 (2)	0.102 (3)	
F10	−0.1663 (8)	0.9136 (6)	0.5896 (3)	0.138 (5)	
F11	−0.0222 (8)	0.9118 (6)	0.6053 (4)	0.155 (6)	
F12	−0.0856 (8)	0.8756 (6)	0.5562 (3)	0.124 (4)	
O1	0.5156 (6)	0.5946 (5)	0.6766 (2)	0.068 (3)	
O2	0.4553 (7)	0.5785 (5)	0.7210 (2)	0.069 (3)	
O3	0.4065 (7)	0.5502 (4)	0.6054 (2)	0.052 (3)	
O4	0.1719 (6)	1.0856 (5)	0.6740 (2)	0.058 (3)	
O5	0.2495 (6)	1.0998 (4)	0.7245 (2)	0.055 (2)	
O6	0.2005 (7)	1.0504 (4)	0.6045 (2)	0.052 (2)	
O11	−0.5063 (8)	0.9228 (4)	0.6140 (2)	0.058 (3)	
O12	−0.5574 (7)	1.0292 (5)	0.6202 (2)	0.063 (3)	
O13	−0.4143 (8)	0.9922 (5)	0.6546 (2)	0.081 (3)	
O24	−0.2636 (12)	1.3026 (9)	0.6384 (3)	0.173 (7)	
O25	−0.3926 (7)	1.2725 (5)	0.5971 (3)	0.077 (3)	
O26	−0.2361 (7)	1.2697 (5)	0.5856 (3)	0.074 (3)	
O37	0.0665 (7)	0.4897 (4)	0.6528 (2)	0.062 (3)	
O38	0.1066 (7)	0.4178 (5)	0.6116 (2)	0.057 (3)	
O39	0.1759 (7)	0.5261 (4)	0.6192 (2)	0.059 (3)	
O41	−0.1808 (6)	0.7739 (5)	0.5878 (3)	0.068 (3)	
O42	−0.1049 (11)	0.8135 (10)	0.6402 (3)	0.170 (7)	
O43	−0.0137 (7)	0.7720 (5)	0.6010 (3)	0.091 (4)	
S1	−0.4791 (3)	0.98590 (16)	0.62398 (9)	0.0493 (9)	
S2	−0.2991 (3)	1.2956 (2)	0.60518 (10)	0.0590 (11)	
S3	0.0996 (2)	0.48376 (16)	0.62287 (9)	0.0463 (8)	
S4	−0.0973 (3)	0.8004 (2)	0.60562 (10)	0.0632 (11)	
C01	−0.4118 (10)	1.0139 (8)	0.5938 (4)	0.058 (4)	
C02	−0.3090 (15)	1.3795 (11)	0.5891 (6)	0.115 (9)	
C03	0.0084 (11)	0.5132 (7)	0.5911 (4)	0.065 (4)	
C04	−0.0904 (12)	0.8770 (9)	0.5895 (5)	0.087 (6)	
C25	−0.258 (3)	1.3744 (18)	0.7055 (9)	0.104 (14)	0.5
H25A	−0.2828	1.4078	0.6898	0.124*	0.5
H25B	−0.2827	1.3338	0.6960	0.124*	0.5
Cl1	−0.1437 (7)	1.3725 (7)	0.7065 (4)	0.166 (6)	0.5
Cl2	−0.3028 (9)	1.3858 (4)	0.7381 (3)	0.119 (4)	0.5
C25A	−0.217 (4)	1.391 (6)	0.6992 (10)	0.08 (3)*	0.1667
H25C	−0.1934	1.4273	0.6884	0.100*	0.1667
H25D	−0.2185	1.3535	0.6848	0.100*	0.1667
Cl1A	−0.146 (2)	1.3760 (18)	0.7359 (9)	0.133 (12)*	0.1667
Cl2A	−0.327 (2)	1.407 (2)	0.7059 (12)	0.153 (15)*	0.1667
O21	0.1377 (12)	0.4568 (7)	0.7890 (4)	0.159 (5)	
O20	0.0258 (15)	0.5096 (11)	0.7639 (6)	0.224 (12)	
C20	0.0559 (15)	0.4743 (13)	0.7793 (8)	0.142 (13)	
C22	0.2052 (17)	0.4973 (11)	0.7785 (6)	0.159 (5)	
H22A	0.1802	0.5407	0.7785	0.191*	
H22B	0.2610	0.4959	0.7957	0.191*	
C21	−0.0054 (18)	0.4212 (12)	0.7971 (7)	0.185 (13)	
H21A	0.0375	0.3923	0.8106	0.278*	
H21B	−0.0433	0.4433	0.8107	0.278*	
H21C	−0.0459	0.3970	0.7803	0.278*	
C23	0.2360 (18)	0.4898 (12)	0.7492 (6)	0.159 (5)	
H23A	0.2818	0.5226	0.7471	0.238*	
H23B	0.2648	0.4481	0.7487	0.238*	
H23C	0.1833	0.4933	0.7313	0.238*	

### [Bis(diphenylphosphanyl)methane]carbonyl({[(diphenylphosphanyl)methyl]diphenylphosphanylidene}(ethoxyoxoethanylidene)methanylidene- κ2P,C}hydridoiridium(III) bis(trifluoromethanesulfonate)–dichloromethane–ethyl acetate (6/2/3) (4) Atomic displacement parameters (Å^2^)

**Table:**

	*U*^11^	*U*^22^	*U*^33^	*U*^12^	*U*^13^	*U*^23^
Ir1	0.0220 (2)	0.0245 (3)	0.0279 (2)	−0.0044 (3)	0.00400 (16)	0.0021 (2)
Ir2	0.0242 (2)	0.0246 (3)	0.0278 (2)	−0.0067 (3)	0.00560 (17)	0.00118 (19)
P1	0.0185 (14)	0.0256 (19)	0.0305 (16)	−0.0062 (12)	0.0027 (12)	0.0003 (12)
P2	0.0279 (17)	0.0316 (19)	0.0289 (17)	−0.0029 (15)	0.0077 (13)	0.0019 (13)
P3	0.0265 (15)	0.0307 (18)	0.0255 (15)	−0.0085 (13)	0.0054 (12)	0.0002 (13)
P4	0.0227 (15)	0.037 (2)	0.0325 (16)	−0.0036 (14)	0.0041 (13)	0.0045 (13)
P5	0.0266 (15)	0.0283 (19)	0.0306 (17)	−0.0031 (13)	0.0051 (13)	0.0027 (13)
P6	0.0265 (17)	0.0276 (18)	0.0334 (17)	−0.0070 (15)	0.0034 (13)	0.0015 (14)
P7	0.0204 (14)	0.0317 (18)	0.0323 (17)	−0.0047 (13)	0.0037 (12)	0.0026 (13)
P8	0.0268 (15)	0.034 (2)	0.0348 (17)	−0.0102 (14)	0.0120 (13)	−0.0014 (13)
C1	0.039 (7)	0.022 (8)	0.021 (6)	−0.009 (5)	0.004 (5)	−0.004 (5)
C2	0.035 (6)	0.027 (7)	0.039 (6)	−0.015 (6)	0.007 (5)	−0.001 (5)
C3	0.026 (6)	0.029 (7)	0.043 (7)	−0.017 (5)	−0.002 (5)	0.000 (6)
C4	0.026 (6)	0.018 (7)	0.055 (8)	−0.001 (5)	0.008 (6)	0.007 (6)
C5	0.047 (8)	0.053 (9)	0.036 (8)	0.000 (6)	0.004 (6)	0.012 (6)
C6	0.055 (9)	0.101 (13)	0.101 (13)	0.043 (9)	0.013 (9)	0.069 (11)
C7	0.17 (2)	0.18 (2)	0.18 (2)	0.138 (19)	0.099 (18)	0.139 (19)
C8	0.022 (6)	0.045 (10)	0.036 (7)	0.001 (6)	0.007 (5)	−0.002 (6)
C11	0.028 (6)	0.028 (8)	0.019 (5)	−0.016 (6)	0.007 (5)	−0.001 (5)
C12	0.019 (5)	0.032 (7)	0.038 (6)	−0.011 (6)	0.001 (4)	0.006 (5)
C13	0.034 (7)	0.043 (8)	0.029 (6)	−0.008 (6)	0.002 (5)	−0.002 (6)
C14	0.035 (7)	0.028 (7)	0.034 (7)	−0.003 (5)	0.002 (5)	0.002 (5)
C15	0.041 (8)	0.040 (8)	0.036 (7)	−0.009 (6)	0.011 (6)	−0.002 (6)
C16	0.102 (13)	0.085 (12)	0.050 (9)	−0.024 (10)	0.019 (9)	0.035 (8)
C17	0.133 (16)	0.124 (16)	0.065 (11)	−0.037 (13)	0.041 (11)	0.025 (10)
C18	0.034 (7)	0.025 (8)	0.028 (6)	0.007 (6)	0.012 (5)	0.010 (5)
C101	0.014 (6)	0.052 (9)	0.025 (6)	−0.005 (5)	0.003 (5)	0.004 (5)
C102	0.047 (9)	0.032 (9)	0.042 (8)	−0.007 (7)	0.002 (7)	−0.001 (6)
C103	0.047 (8)	0.018 (7)	0.052 (9)	−0.009 (6)	0.002 (7)	−0.016 (6)
C104	0.051 (8)	0.064 (10)	0.035 (8)	0.001 (7)	0.008 (6)	−0.028 (7)
C105	0.071 (10)	0.059 (11)	0.035 (8)	−0.006 (8)	0.005 (7)	0.001 (7)
C106	0.038 (6)	0.034 (7)	0.032 (6)	0.011 (6)	0.005 (5)	0.007 (6)
C107	0.057 (8)	0.027 (7)	0.023 (6)	−0.012 (7)	0.004 (5)	0.007 (6)
C108	0.022 (6)	0.033 (8)	0.049 (8)	−0.002 (5)	0.008 (5)	0.010 (6)
C109	0.054 (9)	0.026 (8)	0.055 (9)	−0.013 (7)	0.013 (7)	0.017 (6)
C110	0.038 (8)	0.025 (8)	0.083 (10)	0.001 (6)	−0.001 (7)	0.018 (7)
C111	0.045 (8)	0.040 (9)	0.075 (10)	0.008 (7)	−0.012 (7)	0.014 (7)
C112	0.040 (7)	0.037 (8)	0.044 (7)	−0.015 (6)	−0.001 (6)	0.004 (6)
C201	0.022 (5)	0.029 (7)	0.050 (7)	−0.016 (6)	0.012 (5)	0.006 (6)
C202	0.034 (7)	0.058 (10)	0.037 (7)	−0.013 (6)	0.004 (6)	0.007 (6)
C203	0.059 (9)	0.052 (10)	0.057 (9)	−0.026 (7)	0.017 (7)	0.014 (7)
C204	0.064 (9)	0.065 (11)	0.049 (9)	−0.008 (8)	0.028 (8)	0.006 (8)
C205	0.061 (9)	0.076 (13)	0.066 (10)	−0.020 (9)	0.044 (8)	−0.016 (8)
C206	0.028 (7)	0.054 (9)	0.050 (8)	−0.002 (6)	0.018 (6)	−0.008 (7)
C207	0.031 (6)	0.041 (7)	0.021 (5)	−0.014 (6)	0.012 (5)	0.006 (6)
C208	0.032 (7)	0.029 (8)	0.058 (9)	0.001 (5)	0.008 (6)	−0.004 (6)
C209	0.054 (9)	0.055 (10)	0.042 (8)	−0.009 (7)	0.000 (7)	0.004 (7)
C210	0.051 (9)	0.031 (9)	0.064 (10)	−0.002 (7)	0.003 (7)	−0.004 (7)
C211	0.045 (8)	0.032 (9)	0.074 (10)	0.003 (7)	0.021 (8)	0.001 (7)
C212	0.039 (7)	0.012 (7)	0.061 (8)	−0.007 (5)	−0.010 (6)	0.021 (5)
C301	0.027 (6)	0.030 (8)	0.033 (7)	0.001 (5)	0.005 (5)	0.005 (5)
C302	0.046 (8)	0.061 (10)	0.025 (8)	−0.012 (8)	0.001 (6)	−0.009 (8)
C303	0.051 (9)	0.107 (16)	0.032 (8)	0.008 (10)	−0.004 (6)	0.008 (8)
C304	0.054 (10)	0.076 (13)	0.068 (12)	−0.011 (9)	0.008 (8)	0.036 (9)
C305	0.072 (11)	0.059 (11)	0.061 (10)	−0.009 (9)	0.004 (8)	0.022 (9)
C306	0.048 (8)	0.049 (10)	0.026 (7)	−0.010 (7)	0.007 (6)	0.004 (6)
C307	0.029 (6)	0.047 (8)	0.028 (7)	−0.002 (6)	0.009 (5)	−0.005 (6)
C308	0.067 (9)	0.034 (9)	0.039 (8)	0.000 (7)	0.024 (7)	0.000 (6)
C309	0.108 (13)	0.037 (9)	0.059 (10)	−0.010 (9)	0.056 (10)	−0.019 (7)
C310	0.15 (2)	0.040 (11)	0.076 (12)	0.051 (12)	0.048 (13)	0.007 (9)
C311	0.091 (16)	0.074 (16)	0.085 (15)	0.007 (13)	0.049 (13)	0.008 (12)
C312	0.030 (7)	0.049 (9)	0.065 (9)	−0.001 (6)	0.015 (6)	0.012 (7)
C401	0.029 (7)	0.023 (7)	0.045 (8)	−0.008 (5)	0.007 (6)	−0.010 (6)
C402	0.065 (9)	0.037 (9)	0.023 (6)	−0.017 (6)	−0.011 (6)	−0.003 (5)
C403	0.066 (10)	0.046 (10)	0.064 (10)	−0.017 (8)	0.010 (8)	−0.012 (8)
C404	0.080 (11)	0.075 (13)	0.034 (8)	−0.014 (9)	−0.015 (8)	0.003 (7)
C405	0.118 (14)	0.039 (11)	0.040 (9)	−0.008 (9)	−0.003 (9)	0.013 (7)
C406	0.077 (10)	0.026 (8)	0.029 (7)	0.010 (7)	0.000 (7)	0.003 (6)
C407	0.048 (8)	0.042 (9)	0.025 (6)	0.007 (6)	0.010 (6)	0.001 (5)
C408	0.042 (7)	0.028 (8)	0.057 (8)	0.009 (6)	0.018 (6)	−0.007 (6)
C409	0.070 (11)	0.058 (11)	0.062 (10)	0.028 (9)	0.019 (9)	−0.002 (8)
C410	0.034 (9)	0.119 (16)	0.064 (10)	0.038 (9)	0.016 (7)	0.023 (11)
C411	0.032 (9)	0.102 (16)	0.101 (14)	0.046 (10)	0.026 (8)	0.022 (12)
C412	0.034 (8)	0.101 (13)	0.050 (8)	−0.002 (9)	0.002 (6)	0.023 (9)
C501	0.030 (7)	0.032 (8)	0.031 (7)	−0.009 (5)	0.007 (5)	−0.001 (5)
C502	0.040 (7)	0.037 (8)	0.045 (7)	0.002 (7)	0.003 (6)	−0.001 (7)
C503	0.061 (9)	0.072 (11)	0.028 (7)	−0.011 (8)	0.010 (7)	−0.008 (7)
C504	0.059 (9)	0.056 (11)	0.051 (9)	−0.019 (8)	0.012 (7)	−0.009 (8)
C505	0.058 (9)	0.043 (9)	0.057 (10)	−0.014 (7)	0.017 (8)	−0.003 (7)
C506	0.031 (8)	0.028 (9)	0.040 (8)	−0.010 (6)	0.022 (7)	−0.010 (6)
C507	0.024 (6)	0.025 (7)	0.039 (6)	−0.009 (5)	0.002 (5)	0.007 (6)
C508	0.043 (7)	0.026 (8)	0.053 (8)	−0.012 (6)	0.028 (6)	−0.007 (6)
C509	0.054 (8)	0.016 (8)	0.067 (9)	0.000 (6)	0.017 (7)	0.007 (6)
C510	0.064 (10)	0.035 (9)	0.064 (9)	−0.008 (8)	0.016 (8)	0.011 (7)
C511	0.041 (8)	0.023 (8)	0.083 (10)	−0.005 (6)	0.023 (7)	0.018 (7)
C512	0.035 (7)	0.028 (8)	0.068 (8)	−0.003 (6)	0.024 (6)	0.002 (6)
C601	0.037 (6)	0.023 (7)	0.032 (6)	−0.007 (6)	−0.002 (5)	−0.002 (5)
C602	0.043 (8)	0.038 (8)	0.044 (8)	−0.005 (6)	−0.008 (6)	0.006 (6)
C603	0.032 (7)	0.044 (9)	0.073 (10)	0.006 (6)	−0.003 (7)	0.004 (7)
C604	0.031 (7)	0.086 (12)	0.046 (9)	−0.003 (8)	0.000 (6)	0.003 (8)
C605	0.068 (11)	0.062 (11)	0.048 (9)	−0.018 (9)	−0.028 (8)	−0.004 (8)
C606	0.101 (14)	0.027 (9)	0.049 (9)	−0.004 (8)	−0.025 (9)	−0.007 (7)
C607	0.037 (6)	0.034 (7)	0.032 (6)	0.001 (6)	0.001 (5)	−0.006 (6)
C608	0.042 (7)	0.049 (9)	0.026 (7)	−0.011 (6)	0.013 (6)	−0.003 (5)
C609	0.056 (8)	0.017 (8)	0.058 (9)	0.000 (6)	0.007 (7)	−0.005 (6)
C610	0.065 (10)	0.027 (8)	0.060 (9)	−0.001 (7)	0.008 (8)	0.000 (6)
C611	0.072 (10)	0.030 (9)	0.076 (10)	−0.027 (8)	0.027 (9)	0.003 (7)
C612	0.028 (6)	0.045 (9)	0.048 (8)	−0.013 (6)	−0.006 (6)	0.006 (6)
C701	0.019 (6)	0.046 (9)	0.037 (7)	−0.001 (5)	0.004 (5)	0.003 (6)
C702	0.050 (8)	0.038 (9)	0.063 (12)	−0.019 (8)	0.018 (8)	0.009 (8)
C703	0.072 (10)	0.051 (11)	0.038 (8)	−0.017 (8)	0.010 (7)	−0.006 (7)
C704	0.030 (7)	0.084 (13)	0.049 (9)	−0.021 (7)	0.011 (6)	0.024 (8)
C705	0.058 (9)	0.037 (9)	0.058 (9)	0.013 (7)	0.009 (7)	0.004 (7)
C706	0.061 (9)	0.047 (10)	0.036 (8)	0.000 (7)	0.012 (7)	0.010 (7)
C707	0.029 (6)	0.018 (7)	0.041 (7)	−0.005 (5)	0.000 (5)	0.010 (5)
C708	0.047 (8)	0.040 (9)	0.043 (8)	−0.006 (7)	−0.004 (6)	−0.005 (6)
C709	0.075 (11)	0.039 (10)	0.074 (11)	−0.012 (8)	−0.010 (9)	−0.017 (8)
C710	0.061 (11)	0.049 (11)	0.073 (11)	−0.028 (8)	−0.020 (8)	−0.008 (8)
C711	0.032 (9)	0.085 (15)	0.075 (12)	−0.044 (10)	0.012 (9)	−0.001 (10)
C712	0.051 (9)	0.056 (10)	0.052 (9)	−0.020 (7)	0.001 (7)	−0.002 (7)
C801	0.027 (7)	0.053 (10)	0.048 (8)	−0.007 (6)	0.023 (6)	−0.001 (7)
C802	0.034 (8)	0.065 (10)	0.046 (9)	0.008 (7)	0.016 (7)	0.005 (7)
C803	0.058 (10)	0.079 (13)	0.040 (9)	−0.002 (8)	0.015 (7)	0.005 (8)
C804	0.060 (9)	0.073 (13)	0.060 (10)	−0.025 (8)	0.017 (8)	−0.036 (8)
C805	0.075 (11)	0.061 (11)	0.051 (9)	−0.023 (8)	0.029 (8)	−0.019 (8)
C806	0.040 (8)	0.054 (10)	0.057 (9)	−0.014 (6)	0.017 (7)	0.000 (7)
C807	0.027 (7)	0.047 (9)	0.044 (7)	−0.017 (6)	0.015 (6)	0.004 (6)
C808	0.052 (9)	0.066 (11)	0.049 (8)	−0.026 (8)	0.020 (7)	0.006 (7)
C809	0.088 (13)	0.044 (10)	0.073 (11)	−0.019 (10)	−0.001 (10)	0.000 (8)
C810	0.054 (11)	0.105 (16)	0.097 (14)	−0.040 (11)	0.001 (10)	0.031 (12)
C811	0.042 (10)	0.114 (17)	0.085 (13)	−0.015 (11)	0.033 (9)	0.008 (12)
C812	0.040 (8)	0.060 (11)	0.072 (10)	−0.014 (7)	0.027 (7)	−0.006 (8)
F1	0.105 (8)	0.060 (7)	0.117 (8)	−0.042 (6)	0.024 (6)	0.011 (6)
F2	0.063 (6)	0.104 (8)	0.144 (10)	0.008 (6)	0.041 (6)	0.013 (7)
F3	0.101 (7)	0.086 (7)	0.066 (6)	−0.020 (6)	0.024 (6)	0.010 (5)
F4	0.147 (12)	0.090 (9)	0.194 (14)	0.016 (9)	0.071 (11)	0.033 (9)
F5	0.132 (11)	0.099 (10)	0.42 (3)	−0.090 (9)	0.161 (14)	−0.104 (12)
F6	0.140 (11)	0.070 (8)	0.215 (14)	−0.017 (8)	0.088 (10)	−0.039 (8)
F7	0.091 (7)	0.096 (7)	0.046 (5)	0.002 (6)	0.016 (5)	0.001 (5)
F8	0.091 (7)	0.064 (7)	0.111 (8)	0.027 (6)	0.030 (6)	0.017 (6)
F9	0.068 (6)	0.122 (9)	0.113 (8)	−0.039 (6)	0.008 (6)	0.004 (7)
F10	0.079 (8)	0.124 (10)	0.200 (14)	0.028 (7)	−0.003 (8)	−0.028 (9)
F11	0.062 (7)	0.085 (9)	0.279 (17)	−0.014 (6)	−0.075 (9)	−0.009 (9)
F12	0.098 (8)	0.135 (10)	0.131 (10)	−0.026 (8)	0.002 (8)	0.026 (8)
O1	0.053 (6)	0.084 (8)	0.072 (7)	0.034 (5)	0.027 (5)	0.033 (6)
O2	0.074 (7)	0.087 (8)	0.051 (6)	0.037 (6)	0.028 (5)	0.041 (5)
O3	0.067 (7)	0.027 (6)	0.063 (6)	−0.012 (5)	0.013 (5)	0.003 (5)
O4	0.055 (6)	0.065 (7)	0.052 (6)	−0.017 (5)	0.006 (5)	0.008 (5)
O5	0.050 (5)	0.077 (7)	0.040 (5)	−0.022 (5)	0.012 (4)	0.014 (4)
O6	0.074 (7)	0.023 (6)	0.061 (6)	−0.003 (5)	0.021 (5)	0.004 (5)
O11	0.080 (8)	0.022 (6)	0.072 (7)	−0.003 (5)	0.013 (6)	0.003 (5)
O12	0.051 (6)	0.057 (7)	0.079 (7)	0.004 (5)	0.007 (6)	0.007 (5)
O13	0.116 (9)	0.063 (7)	0.051 (6)	−0.015 (7)	−0.022 (6)	0.007 (5)
O24	0.182 (15)	0.265 (19)	0.061 (8)	0.051 (16)	−0.013 (9)	−0.066 (11)
O25	0.041 (6)	0.089 (8)	0.104 (8)	−0.011 (5)	0.015 (6)	−0.006 (7)
O26	0.064 (7)	0.087 (8)	0.077 (7)	0.006 (6)	0.032 (6)	−0.009 (6)
O37	0.097 (8)	0.045 (6)	0.051 (6)	−0.020 (5)	0.031 (6)	−0.002 (4)
O38	0.069 (7)	0.040 (7)	0.066 (7)	−0.005 (5)	0.023 (5)	0.001 (5)
O39	0.055 (6)	0.042 (6)	0.081 (7)	−0.018 (5)	0.019 (6)	0.002 (5)
O41	0.028 (5)	0.086 (8)	0.089 (8)	−0.026 (5)	0.009 (5)	0.002 (6)
O42	0.159 (14)	0.27 (2)	0.084 (10)	−0.073 (15)	0.024 (10)	−0.022 (12)
O43	0.039 (6)	0.056 (7)	0.169 (11)	0.015 (5)	−0.008 (7)	−0.023 (7)
S1	0.060 (2)	0.034 (2)	0.048 (2)	−0.0074 (18)	−0.0062 (18)	0.0035 (16)
S2	0.048 (2)	0.074 (3)	0.056 (2)	−0.009 (2)	0.013 (2)	−0.017 (2)
S3	0.053 (2)	0.036 (2)	0.053 (2)	−0.0073 (17)	0.0169 (17)	0.0012 (17)
S4	0.042 (2)	0.080 (3)	0.065 (2)	−0.015 (2)	0.0044 (19)	−0.013 (3)
C01	0.046 (9)	0.074 (12)	0.053 (10)	−0.004 (8)	0.007 (7)	0.006 (8)
C02	0.081 (15)	0.104 (18)	0.16 (2)	0.013 (13)	0.035 (15)	−0.098 (18)
C03	0.081 (12)	0.027 (9)	0.092 (13)	−0.003 (8)	0.026 (10)	0.000 (8)
C04	0.052 (11)	0.086 (14)	0.114 (16)	−0.007 (10)	−0.010 (11)	−0.002 (12)
C25	0.09 (3)	0.11 (3)	0.13 (3)	−0.05 (2)	0.08 (3)	−0.04 (2)
Cl1	0.069 (7)	0.239 (16)	0.183 (13)	−0.009 (8)	0.001 (7)	−0.094 (12)
Cl2	0.201 (11)	0.064 (6)	0.109 (8)	−0.012 (7)	0.077 (8)	−0.033 (5)
O21	0.147 (12)	0.111 (10)	0.207 (14)	−0.023 (7)	0.000 (10)	−0.074 (10)
O20	0.19 (2)	0.23 (2)	0.29 (3)	0.108 (18)	0.143 (19)	0.14 (2)
C20	0.066 (13)	0.14 (2)	0.20 (3)	0.057 (15)	−0.036 (16)	−0.09 (2)
C22	0.147 (12)	0.111 (10)	0.207 (14)	−0.023 (7)	0.000 (10)	−0.074 (10)
C21	0.18 (3)	0.15 (2)	0.27 (3)	0.02 (2)	0.15 (3)	0.06 (2)
C23	0.147 (12)	0.111 (10)	0.207 (14)	−0.023 (7)	0.000 (10)	−0.074 (10)

### [Bis(diphenylphosphanyl)methane]carbonyl({[(diphenylphosphanyl)methyl]diphenylphosphanylidene}(ethoxyoxoethanylidene)methanylidene- κ2P,C}hydridoiridium(III) bis(trifluoromethanesulfonate)–dichloromethane–ethyl acetate (6/2/3) (4) Geometric parameters (Å, º)

**Table:**

Ir1—C8	1.965 (15)	C406—H406	0.9400
Ir1—C1	2.131 (11)	C407—C408	1.363 (16)
Ir1—P1	2.334 (3)	C407—C412	1.435 (17)
Ir1—P4	2.377 (3)	C408—C409	1.412 (16)
Ir1—P3	2.379 (3)	C408—H408	0.9400
Ir1—H1	1.60 (2)	C409—C410	1.40 (2)
Ir2—C18	1.962 (13)	C409—H409	0.9400
Ir2—C11	2.152 (10)	C410—C411	1.38 (2)
Ir2—P5	2.340 (3)	C410—H410	0.9400
Ir2—P7	2.360 (3)	C411—C412	1.43 (2)
Ir2—P8	2.377 (3)	C411—H411	0.9400
Ir2—H2	1.59 (2)	C412—H412	0.9400
P1—C101	1.784 (11)	C501—C502	1.376 (15)
P1—C107	1.811 (13)	C501—C506	1.406 (16)
P1—C2	1.844 (10)	C502—C503	1.392 (16)
P2—C207	1.778 (13)	C502—H502	0.9400
P2—C2	1.787 (10)	C503—C504	1.350 (18)
P2—C201	1.803 (10)	C503—H503	0.9400
P2—C1	1.826 (12)	C504—C505	1.369 (18)
P3—C301	1.796 (11)	C504—H504	0.9400
P3—C307	1.803 (12)	C505—C506	1.376 (17)
P3—C3	1.825 (10)	C505—H505	0.9400
P4—C401	1.789 (11)	C506—H506	0.9400
P4—C3	1.837 (11)	C507—C512	1.418 (14)
P4—C407	1.849 (12)	C507—C508	1.420 (16)
P5—C507	1.776 (12)	C508—C509	1.369 (16)
P5—C12	1.830 (10)	C508—H508	0.9400
P5—C501	1.832 (11)	C509—C510	1.380 (18)
P6—C11	1.771 (11)	C509—H509	0.9400
P6—C12	1.776 (11)	C510—C511	1.325 (17)
P6—C601	1.789 (11)	C510—H510	0.9400
P6—C607	1.797 (13)	C511—C512	1.402 (16)
P7—C707	1.806 (11)	C511—H511	0.9400
P7—C701	1.809 (12)	C512—H512	0.9400
P7—C13	1.833 (11)	C601—C606	1.370 (16)
P7—P8	2.698 (4)	C601—C602	1.379 (16)
P8—C807	1.802 (11)	C602—C603	1.376 (16)
P8—C801	1.820 (13)	C602—H602	0.9400
P8—C13	1.823 (11)	C603—C604	1.353 (18)
C1—C4	1.342 (15)	C603—H603	0.9400
C2—H2A	0.9800	C604—C605	1.359 (18)
C2—H2B	0.9800	C604—H604	0.9400
C3—H3A	0.9800	C605—C606	1.343 (19)
C3—H3B	0.9800	C605—H605	0.9400
C4—C5	1.461 (16)	C606—H606	0.9400
C4—H4	0.9400	C607—C608	1.398 (15)
C5—O1	1.190 (14)	C607—C612	1.413 (16)
C5—O2	1.323 (13)	C608—C609	1.362 (15)
C6—C7	1.36 (2)	C608—H608	0.9400
C6—O2	1.442 (14)	C609—C610	1.366 (17)
C6—H6A	0.9800	C609—H609	0.9400
C6—H6B	0.9800	C610—C611	1.376 (18)
C7—H7A	0.9700	C610—H610	0.9400
C7—H7B	0.9700	C611—C612	1.393 (17)
C7—H7C	0.9700	C611—H611	0.9400
C8—O3	1.116 (14)	C612—H612	0.9400
C11—C14	1.335 (14)	C701—C706	1.386 (16)
C12—H12A	0.9800	C701—C702	1.443 (18)
C12—H12B	0.9800	C702—C703	1.362 (19)
C13—H13A	0.9800	C702—H702	0.9400
C13—H13B	0.9800	C703—C704	1.373 (18)
C14—C15	1.490 (15)	C703—H703	0.9400
C14—H14	0.9400	C704—C705	1.399 (18)
C15—O4	1.173 (13)	C704—H704	0.9400
C15—O5	1.340 (13)	C705—C706	1.365 (17)
C16—O5	1.491 (15)	C705—H705	0.9400
C16—C17	1.502 (19)	C706—H706	0.9400
C16—H16A	0.9800	C707—C708	1.388 (16)
C16—H16B	0.9800	C707—C712	1.397 (17)
C17—H17A	0.9700	C708—C709	1.375 (17)
C17—H17B	0.9700	C708—H708	0.9400
C17—H17C	0.9700	C709—C710	1.36 (2)
C18—O6	1.123 (13)	C709—H709	0.9400
C101—C106	1.394 (14)	C710—C711	1.39 (2)
C101—C102	1.412 (17)	C710—H710	0.9400
C102—C103	1.381 (16)	C711—C712	1.378 (18)
C102—H102	0.9400	C711—H711	0.9400
C103—C104	1.383 (17)	C712—H712	0.9400
C103—H103	0.9400	C801—C802	1.386 (18)
C104—C105	1.383 (17)	C801—C806	1.396 (17)
C104—H104	0.9400	C802—C803	1.334 (18)
C105—C106	1.376 (16)	C802—H802	0.9400
C105—H105	0.9400	C803—C804	1.378 (19)
C106—H106	0.9400	C803—H803	0.9400
C107—C108	1.380 (16)	C804—C805	1.419 (19)
C107—C112	1.400 (15)	C804—H804	0.9400
C108—C109	1.384 (16)	C805—C806	1.364 (17)
C108—H108	0.9400	C805—H805	0.9400
C109—C110	1.399 (17)	C806—H806	0.9400
C109—H109	0.9400	C807—C808	1.355 (17)
C110—C111	1.400 (17)	C807—C812	1.417 (17)
C110—H110	0.9400	C808—C809	1.394 (19)
C111—C112	1.356 (17)	C808—H808	0.9400
C111—H111	0.9400	C809—C810	1.34 (2)
C112—H112	0.9400	C809—H809	0.9400
C201—C206	1.376 (16)	C810—C811	1.43 (2)
C201—C202	1.385 (16)	C810—H810	0.9400
C202—C203	1.390 (16)	C811—C812	1.324 (19)
C202—H202	0.9400	C811—H811	0.9400
C203—C204	1.350 (18)	C812—H812	0.9400
C203—H203	0.9400	F1—C01	1.337 (17)
C204—C205	1.356 (18)	F2—C01	1.292 (16)
C204—H204	0.9400	F3—C01	1.341 (15)
C205—C206	1.433 (17)	F4—C02	1.30 (2)
C205—H205	0.9400	F5—C02	1.21 (2)
C206—H206	0.9400	F6—C02	1.44 (2)
C207—C212	1.376 (15)	F7—C03	1.288 (16)
C207—C208	1.400 (15)	F8—C03	1.344 (16)
C208—C209	1.402 (16)	F9—C03	1.378 (16)
C208—H208	0.9400	F10—C04	1.341 (19)
C209—C210	1.333 (17)	F11—C04	1.306 (19)
C209—H209	0.9400	F12—C04	1.39 (2)
C210—C211	1.359 (17)	O11—S1	1.420 (10)
C210—H210	0.9400	O12—S1	1.438 (10)
C211—C212	1.377 (16)	O13—S1	1.445 (9)
C211—H211	0.9400	O24—S2	1.390 (11)
C212—H212	0.9400	O25—S2	1.421 (10)
C301—C302	1.362 (15)	O26—S2	1.427 (10)
C301—C306	1.385 (15)	O37—S3	1.409 (9)
C302—C303	1.396 (19)	O38—S3	1.468 (10)
C302—H302	0.9400	O39—S3	1.444 (9)
C303—C304	1.35 (2)	O41—S4	1.414 (9)
C303—H303	0.9400	O42—S4	1.480 (13)
C304—C305	1.36 (2)	O43—S4	1.391 (10)
C304—H304	0.9400	S1—C01	1.811 (15)
C305—C306	1.401 (17)	S2—C02	1.88 (3)
C305—H305	0.9400	S3—C03	1.801 (16)
C306—H306	0.9400	S4—C04	1.749 (19)
C307—C308	1.394 (17)	C25—Cl2	1.62 (3)
C307—C312	1.415 (16)	C25—Cl1	1.64 (4)
C308—C309	1.362 (17)	C25—H25A	0.9800
C308—H308	0.9400	C25—H25B	0.9800
C309—C310	1.39 (2)	C25A—Cl2A	1.70 (2)
C309—H309	0.9400	C25A—Cl1A	1.70 (2)
C310—C311	1.34 (2)	C25A—H25C	0.9800
C310—H310	0.9400	C25A—H25D	0.9800
C311—C312	1.36 (2)	O21—C20	1.24 (2)
C311—H311	0.9400	O21—C22	1.42 (3)
C312—H312	0.9400	O20—C20	1.02 (3)
C401—C402	1.384 (16)	C20—C21	1.67 (4)
C401—C406	1.392 (17)	C22—C23	1.37 (3)
C402—C403	1.404 (16)	C22—H22A	0.9800
C402—H402	0.9400	C22—H22B	0.9800
C403—C404	1.353 (18)	C21—H21A	0.9700
C403—H403	0.9400	C21—H21B	0.9700
C404—C405	1.323 (18)	C21—H21C	0.9700
C404—H404	0.9400	C23—H23A	0.9700
C405—C406	1.397 (18)	C23—H23B	0.9700
C405—H405	0.9400	C23—H23C	0.9700
			
C8—Ir1—C1	88.7 (5)	C402—C401—P4	120.9 (10)
C8—Ir1—P1	96.5 (4)	C406—C401—P4	120.3 (9)
C1—Ir1—P1	88.5 (3)	C401—C402—C403	119.6 (12)
C8—Ir1—P4	102.6 (4)	C401—C402—H402	120.2
C1—Ir1—P4	99.8 (3)	C403—C402—H402	120.2
P1—Ir1—P4	159.28 (10)	C404—C403—C402	120.6 (14)
C8—Ir1—P3	99.8 (4)	C404—C403—H403	119.7
C1—Ir1—P3	167.3 (3)	C402—C403—H403	119.7
P1—Ir1—P3	99.83 (9)	C405—C404—C403	119.8 (15)
P4—Ir1—P3	69.25 (9)	C405—C404—H404	120.1
C8—Ir1—H1	168 (4)	C403—C404—H404	120.1
C1—Ir1—H1	103 (4)	C404—C405—C406	122.3 (14)
P1—Ir1—H1	80 (4)	C404—C405—H405	118.8
P4—Ir1—H1	79 (4)	C406—C405—H405	118.8
P3—Ir1—H1	70 (4)	C401—C406—C405	118.5 (13)
C18—Ir2—C11	87.9 (5)	C401—C406—H406	120.7
C18—Ir2—P5	94.7 (3)	C405—C406—H406	120.7
C11—Ir2—P5	87.5 (3)	C408—C407—C412	125.3 (13)
C18—Ir2—P7	101.2 (4)	C408—C407—P4	123.8 (10)
C11—Ir2—P7	167.8 (3)	C412—C407—P4	110.8 (10)
P5—Ir2—P7	99.60 (10)	C407—C408—C409	120.0 (13)
C18—Ir2—P8	104.9 (4)	C407—C408—H408	120.0
C11—Ir2—P8	100.6 (3)	C409—C408—H408	120.0
P5—Ir2—P8	158.91 (11)	C410—C409—C408	116.7 (14)
P7—Ir2—P8	69.44 (10)	C410—C409—H409	121.7
C18—Ir2—H2	174 (4)	C408—C409—H409	121.7
C11—Ir2—H2	92 (4)	C411—C410—C409	123.0 (14)
P5—Ir2—H2	91 (4)	C411—C410—H410	118.5
P7—Ir2—H2	78 (4)	C409—C410—H410	118.5
P8—Ir2—H2	69 (4)	C410—C411—C412	122.2 (16)
C101—P1—C107	106.0 (5)	C410—C411—H411	118.9
C101—P1—C2	106.7 (5)	C412—C411—H411	118.9
C107—P1—C2	103.4 (6)	C411—C412—C407	112.8 (16)
C101—P1—Ir1	115.8 (4)	C411—C412—H412	123.6
C107—P1—Ir1	117.1 (4)	C407—C412—H412	123.6
C2—P1—Ir1	106.6 (3)	C502—C501—C506	118.6 (11)
C207—P2—C2	110.7 (6)	C502—C501—P5	120.9 (9)
C207—P2—C201	108.2 (5)	C506—C501—P5	120.3 (9)
C2—P2—C201	110.0 (5)	C501—C502—C503	120.7 (13)
C207—P2—C1	110.6 (5)	C501—C502—H502	119.6
C2—P2—C1	103.5 (5)	C503—C502—H502	119.6
C201—P2—C1	113.9 (5)	C504—C503—C502	119.7 (13)
C301—P3—C307	104.3 (5)	C504—C503—H503	120.2
C301—P3—C3	111.1 (5)	C502—C503—H503	120.2
C307—P3—C3	107.7 (5)	C503—C504—C505	120.9 (13)
C301—P3—Ir1	126.5 (4)	C503—C504—H504	119.5
C307—P3—Ir1	113.7 (4)	C505—C504—H504	119.5
C3—P3—Ir1	92.0 (4)	C504—C505—C506	120.5 (14)
C401—P4—C3	109.5 (5)	C504—C505—H505	119.8
C401—P4—C407	104.4 (5)	C506—C505—H505	119.8
C3—P4—C407	101.7 (5)	C505—C506—C501	119.6 (13)
C401—P4—Ir1	119.3 (4)	C505—C506—H506	120.2
C3—P4—Ir1	91.8 (3)	C501—C506—H506	120.2
C407—P4—Ir1	126.5 (4)	C512—C507—C508	116.8 (11)
C507—P5—C12	104.6 (5)	C512—C507—P5	117.8 (9)
C507—P5—C501	105.9 (5)	C508—C507—P5	125.3 (8)
C12—P5—C501	105.6 (5)	C509—C508—C507	121.6 (11)
C507—P5—Ir2	118.1 (4)	C509—C508—H508	119.2
C12—P5—Ir2	104.4 (3)	C507—C508—H508	119.2
C501—P5—Ir2	117.0 (4)	C508—C509—C510	118.9 (13)
C11—P6—C12	101.4 (5)	C508—C509—H509	120.5
C11—P6—C601	114.1 (5)	C510—C509—H509	120.5
C12—P6—C601	110.4 (5)	C511—C510—C509	122.2 (13)
C11—P6—C607	110.1 (6)	C511—C510—H510	118.9
C12—P6—C607	113.3 (6)	C509—C510—H510	118.9
C601—P6—C607	107.6 (5)	C510—C511—C512	120.8 (12)
C707—P7—C701	105.0 (5)	C510—C511—H511	119.6
C707—P7—C13	108.3 (5)	C512—C511—H511	119.6
C701—P7—C13	109.5 (6)	C511—C512—C507	119.5 (12)
C707—P7—Ir2	114.2 (4)	C511—C512—H512	120.2
C701—P7—Ir2	126.4 (4)	C507—C512—H512	120.2
C13—P7—Ir2	91.7 (4)	C606—C601—C602	118.6 (12)
C707—P7—P8	101.5 (4)	C606—C601—P6	123.5 (11)
C701—P7—P8	147.0 (4)	C602—C601—P6	118.0 (8)
C13—P7—P8	42.3 (4)	C603—C602—C601	120.3 (12)
Ir2—P7—P8	55.58 (8)	C603—C602—H602	119.9
C807—P8—C801	105.2 (5)	C601—C602—H602	119.9
C807—P8—C13	103.7 (5)	C604—C603—C602	119.3 (14)
C801—P8—C13	110.3 (6)	C604—C603—H603	120.3
C807—P8—Ir2	125.2 (4)	C602—C603—H603	120.3
C801—P8—Ir2	118.3 (4)	C603—C604—C605	120.4 (13)
C13—P8—Ir2	91.4 (4)	C603—C604—H604	119.8
C807—P8—P7	105.4 (4)	C605—C604—H604	119.8
C801—P8—P7	143.5 (4)	C606—C605—C604	120.4 (14)
C13—P8—P7	42.6 (4)	C606—C605—H605	119.8
Ir2—P8—P7	54.99 (8)	C604—C605—H605	119.8
C4—C1—P2	112.8 (9)	C605—C606—C601	120.7 (14)
C4—C1—Ir1	134.0 (9)	C605—C606—H606	119.7
P2—C1—Ir1	112.4 (5)	C601—C606—H606	119.7
P2—C2—P1	107.6 (5)	C608—C607—C612	120.6 (12)
P2—C2—H2A	110.2	C608—C607—P6	121.2 (9)
P1—C2—H2A	110.2	C612—C607—P6	118.0 (9)
P2—C2—H2B	110.2	C609—C608—C607	119.8 (12)
P1—C2—H2B	110.2	C609—C608—H608	120.1
H2A—C2—H2B	108.5	C607—C608—H608	120.1
P3—C3—P4	95.1 (5)	C608—C609—C610	120.3 (13)
P3—C3—H3A	112.7	C608—C609—H609	119.8
P4—C3—H3A	112.7	C610—C609—H609	119.8
P3—C3—H3B	112.7	C609—C610—C611	120.8 (13)
P4—C3—H3B	112.7	C609—C610—H610	119.6
H3A—C3—H3B	110.2	C611—C610—H610	119.6
C1—C4—C5	126.0 (11)	C610—C611—C612	121.2 (12)
C1—C4—H4	117.0	C610—C611—H611	119.4
C5—C4—H4	117.0	C612—C611—H611	119.4
O1—C5—O2	121.7 (12)	C611—C612—C607	117.0 (13)
O1—C5—C4	127.6 (11)	C611—C612—H612	121.5
O2—C5—C4	110.2 (11)	C607—C612—H612	121.5
C7—C6—O2	111.9 (13)	C706—C701—C702	119.0 (12)
C7—C6—H6A	109.2	C706—C701—P7	123.0 (10)
O2—C6—H6A	109.2	C702—C701—P7	118.0 (10)
C7—C6—H6B	109.2	C703—C702—C701	118.5 (14)
O2—C6—H6B	109.2	C703—C702—H702	120.8
H6A—C6—H6B	107.9	C701—C702—H702	120.8
C6—C7—H7A	109.5	C702—C703—C704	122.3 (14)
C6—C7—H7B	109.5	C702—C703—H703	118.8
H7A—C7—H7B	109.5	C704—C703—H703	118.8
C6—C7—H7C	109.5	C703—C704—C705	118.8 (13)
H7A—C7—H7C	109.5	C703—C704—H704	120.6
H7B—C7—H7C	109.5	C705—C704—H704	120.6
O3—C8—Ir1	175.8 (12)	C706—C705—C704	121.0 (14)
C14—C11—P6	115.4 (8)	C706—C705—H705	119.5
C14—C11—Ir2	132.0 (8)	C704—C705—H705	119.5
P6—C11—Ir2	112.5 (5)	C705—C706—C701	120.3 (13)
P6—C12—P5	108.0 (6)	C705—C706—H706	119.8
P6—C12—H12A	110.1	C701—C706—H706	119.8
P5—C12—H12A	110.1	C708—C707—C712	118.8 (11)
P6—C12—H12B	110.1	C708—C707—P7	120.2 (9)
P5—C12—H12B	110.1	C712—C707—P7	121.0 (10)
H12A—C12—H12B	108.4	C709—C708—C707	120.5 (13)
P8—C13—P7	95.1 (6)	C709—C708—H708	119.8
P8—C13—H13A	112.7	C707—C708—H708	119.8
P7—C13—H13A	112.7	C710—C709—C708	122.1 (15)
P8—C13—H13B	112.7	C710—C709—H709	118.9
P7—C13—H13B	112.7	C708—C709—H709	118.9
H13A—C13—H13B	110.2	C709—C710—C711	117.0 (14)
C11—C14—C15	127.1 (10)	C709—C710—H710	121.5
C11—C14—H14	116.5	C711—C710—H710	121.5
C15—C14—H14	116.5	C712—C711—C710	122.9 (15)
O4—C15—O5	123.4 (11)	C712—C711—H711	118.6
O4—C15—C14	127.2 (11)	C710—C711—H711	118.6
O5—C15—C14	109.4 (10)	C711—C712—C707	118.7 (14)
O5—C16—C17	105.2 (12)	C711—C712—H712	120.7
O5—C16—H16A	110.7	C707—C712—H712	120.7
C17—C16—H16A	110.7	C802—C801—C806	118.5 (13)
O5—C16—H16B	110.7	C802—C801—P8	119.8 (11)
C17—C16—H16B	110.7	C806—C801—P8	121.7 (11)
H16A—C16—H16B	108.8	C803—C802—C801	122.6 (15)
C16—C17—H17A	109.5	C803—C802—H802	118.7
C16—C17—H17B	109.5	C801—C802—H802	118.7
H17A—C17—H17B	109.5	C802—C803—C804	118.7 (15)
C16—C17—H17C	109.5	C802—C803—H803	120.6
H17A—C17—H17C	109.5	C804—C803—H803	120.6
H17B—C17—H17C	109.5	C803—C804—C805	121.0 (13)
O6—C18—Ir2	178.3 (10)	C803—C804—H804	119.5
C106—C101—C102	118.3 (11)	C805—C804—H804	119.5
C106—C101—P1	120.9 (9)	C806—C805—C804	118.1 (14)
C102—C101—P1	120.7 (9)	C806—C805—H805	120.9
C103—C102—C101	119.1 (13)	C804—C805—H805	120.9
C103—C102—H102	120.5	C805—C806—C801	120.7 (14)
C101—C102—H102	120.5	C805—C806—H806	119.7
C102—C103—C104	122.1 (12)	C801—C806—H806	119.7
C102—C103—H103	119.0	C808—C807—C812	117.8 (12)
C104—C103—H103	119.0	C808—C807—P8	124.7 (10)
C105—C104—C103	118.7 (11)	C812—C807—P8	117.5 (11)
C105—C104—H104	120.7	C807—C808—C809	120.1 (14)
C103—C104—H104	120.7	C807—C808—H808	119.9
C106—C105—C104	120.4 (12)	C809—C808—H808	119.9
C106—C105—H105	119.8	C810—C809—C808	120.0 (16)
C104—C105—H105	119.8	C810—C809—H809	120.0
C105—C106—C101	121.4 (13)	C808—C809—H809	120.0
C105—C106—H106	119.3	C809—C810—C811	121.9 (15)
C101—C106—H106	119.3	C809—C810—H810	119.1
C108—C107—C112	117.1 (12)	C811—C810—H810	119.1
C108—C107—P1	124.8 (9)	C812—C811—C810	116.0 (15)
C112—C107—P1	117.8 (9)	C812—C811—H811	122.0
C107—C108—C109	122.0 (12)	C810—C811—H811	122.0
C107—C108—H108	119.0	C811—C812—C807	124.0 (15)
C109—C108—H108	119.0	C811—C812—H812	118.0
C108—C109—C110	120.3 (12)	C807—C812—H812	118.0
C108—C109—H109	119.9	C5—O2—C6	117.4 (11)
C110—C109—H109	119.9	C15—O5—C16	114.6 (10)
C109—C110—C111	117.4 (11)	O11—S1—O12	112.6 (6)
C109—C110—H110	121.3	O11—S1—O13	116.4 (6)
C111—C110—H110	121.3	O12—S1—O13	114.6 (6)
C112—C111—C110	121.4 (12)	O11—S1—C01	104.8 (7)
C112—C111—H111	119.3	O12—S1—C01	103.3 (7)
C110—C111—H111	119.3	O13—S1—C01	103.3 (7)
C111—C112—C107	121.7 (12)	O24—S2—O25	116.1 (9)
C111—C112—H112	119.2	O24—S2—O26	116.0 (9)
C107—C112—H112	119.2	O25—S2—O26	114.4 (7)
C206—C201—C202	119.5 (11)	O24—S2—C02	104.1 (11)
C206—C201—P2	119.6 (10)	O25—S2—C02	103.1 (9)
C202—C201—P2	120.8 (9)	O26—S2—C02	99.9 (8)
C201—C202—C203	119.0 (12)	O37—S3—O39	115.0 (6)
C201—C202—H202	120.5	O37—S3—O38	114.4 (6)
C203—C202—H202	120.5	O39—S3—O38	116.7 (6)
C204—C203—C202	120.8 (13)	O37—S3—C03	106.9 (7)
C204—C203—H203	119.6	O39—S3—C03	100.9 (6)
C202—C203—H203	119.6	O38—S3—C03	100.0 (7)
C203—C204—C205	122.8 (13)	O43—S4—O41	116.4 (7)
C203—C204—H204	118.6	O43—S4—O42	115.2 (9)
C205—C204—H204	118.6	O41—S4—O42	112.0 (8)
C204—C205—C206	116.7 (13)	O43—S4—C04	103.4 (9)
C204—C205—H205	121.6	O41—S4—C04	105.3 (7)
C206—C205—H205	121.6	O42—S4—C04	102.5 (11)
C201—C206—C205	121.1 (13)	F2—C01—F1	108.1 (13)
C201—C206—H206	119.5	F2—C01—F3	107.1 (13)
C205—C206—H206	119.5	F1—C01—F3	105.4 (12)
C212—C207—C208	118.3 (12)	F2—C01—S1	113.7 (11)
C212—C207—P2	122.5 (8)	F1—C01—S1	111.8 (11)
C208—C207—P2	119.1 (9)	F3—C01—S1	110.3 (10)
C207—C208—C209	119.4 (12)	F5—C02—F4	114 (3)
C207—C208—H208	120.3	F5—C02—F6	112.8 (15)
C209—C208—H208	120.3	F4—C02—F6	101.5 (17)
C210—C209—C208	120.1 (13)	F5—C02—S2	114.8 (19)
C210—C209—H209	119.9	F4—C02—S2	107.5 (14)
C208—C209—H209	119.9	F6—C02—S2	104.4 (17)
C209—C210—C211	121.3 (13)	F7—C03—F8	108.5 (12)
C209—C210—H210	119.4	F7—C03—F9	107.2 (13)
C211—C210—H210	119.4	F8—C03—F9	103.7 (13)
C210—C211—C212	120.0 (13)	F7—C03—S3	115.5 (11)
C210—C211—H211	120.0	F8—C03—S3	110.9 (11)
C212—C211—H211	120.0	F9—C03—S3	110.2 (10)
C207—C212—C211	120.7 (12)	F11—C04—F10	103.2 (16)
C207—C212—H212	119.6	F11—C04—F12	110.0 (17)
C211—C212—H212	119.6	F10—C04—F12	101.4 (15)
C302—C301—C306	119.7 (12)	F11—C04—S4	114.3 (14)
C302—C301—P3	120.4 (10)	F10—C04—S4	115.0 (14)
C306—C301—P3	120.0 (9)	F12—C04—S4	111.9 (14)
C301—C302—C303	121.0 (14)	Cl2—C25—Cl1	122 (3)
C301—C302—H302	119.5	Cl2—C25—H25A	106.8
C303—C302—H302	119.5	Cl1—C25—H25A	106.8
C304—C303—C302	117.7 (14)	Cl2—C25—H25B	106.8
C304—C303—H303	121.2	Cl1—C25—H25B	106.8
C302—C303—H303	121.2	H25A—C25—H25B	106.6
C303—C304—C305	123.7 (15)	Cl2A—C25A—Cl1A	109.2 (18)
C303—C304—H304	118.1	Cl2A—C25A—H25C	109.8
C305—C304—H304	118.1	Cl1A—C25A—H25C	109.8
C304—C305—C306	117.7 (15)	Cl2A—C25A—H25D	109.8
C304—C305—H305	121.1	Cl1A—C25A—H25D	109.8
C306—C305—H305	121.1	H25C—C25A—H25D	108.3
C301—C306—C305	120.0 (13)	C20—O21—C22	113 (3)
C301—C306—H306	120.0	O20—C20—O21	134 (4)
C305—C306—H306	120.0	O20—C20—C21	124 (3)
C308—C307—C312	117.3 (12)	O21—C20—C21	102 (3)
C308—C307—P3	121.0 (9)	C23—C22—O21	123 (2)
C312—C307—P3	121.6 (10)	C23—C22—H22A	106.5
C309—C308—C307	121.1 (13)	O21—C22—H22A	106.5
C309—C308—H308	119.4	C23—C22—H22B	106.5
C307—C308—H308	119.4	O21—C22—H22B	106.5
C308—C309—C310	118.1 (15)	H22A—C22—H22B	106.5
C308—C309—H309	120.9	C20—C21—H21A	109.5
C310—C309—H309	120.9	C20—C21—H21B	109.5
C311—C310—C309	123.3 (16)	H21A—C21—H21B	109.5
C311—C310—H310	118.3	C20—C21—H21C	109.5
C309—C310—H310	118.3	H21A—C21—H21C	109.5
C310—C311—C312	118.7 (19)	H21B—C21—H21C	109.5
C310—C311—H311	120.7	C22—C23—H23A	109.5
C312—C311—H311	120.7	C22—C23—H23B	109.5
C311—C312—C307	121.2 (15)	H23A—C23—H23B	109.5
C311—C312—H312	119.4	C22—C23—H23C	109.5
C307—C312—H312	119.4	H23A—C23—H23C	109.5
C402—C401—C406	118.7 (11)	H23B—C23—H23C	109.5
			
C207—P2—C1—C4	113.8 (9)	Ir2—P5—C501—C506	−67.9 (10)
C2—P2—C1—C4	−127.7 (9)	C506—C501—C502—C503	−0.1 (17)
C201—P2—C1—C4	−8.3 (10)	P5—C501—C502—C503	−174.9 (9)
C207—P2—C1—Ir1	−74.6 (6)	C501—C502—C503—C504	1.0 (19)
C2—P2—C1—Ir1	44.0 (7)	C502—C503—C504—C505	−2 (2)
C201—P2—C1—Ir1	163.3 (5)	C503—C504—C505—C506	2 (2)
C207—P2—C2—P1	70.6 (8)	C504—C505—C506—C501	−1 (2)
C201—P2—C2—P1	−169.8 (6)	C502—C501—C506—C505	−0.1 (18)
C1—P2—C2—P1	−47.8 (8)	P5—C501—C506—C505	174.8 (10)
C101—P1—C2—P2	157.6 (6)	C12—P5—C507—C512	64.4 (9)
C107—P1—C2—P2	−90.8 (7)	C501—P5—C507—C512	−46.9 (10)
Ir1—P1—C2—P2	33.3 (8)	Ir2—P5—C507—C512	179.8 (7)
C301—P3—C3—P4	159.2 (5)	C12—P5—C507—C508	−112.6 (10)
C307—P3—C3—P4	−87.2 (6)	C501—P5—C507—C508	136.1 (10)
Ir1—P3—C3—P4	28.5 (4)	Ir2—P5—C507—C508	2.8 (11)
C401—P4—C3—P3	−150.5 (5)	C512—C507—C508—C509	−0.7 (17)
C407—P4—C3—P3	99.4 (6)	P5—C507—C508—C509	176.4 (10)
Ir1—P4—C3—P3	−28.5 (4)	C507—C508—C509—C510	1 (2)
P2—C1—C4—C5	176.1 (10)	C508—C509—C510—C511	−2 (2)
Ir1—C1—C4—C5	6.9 (19)	C509—C510—C511—C512	2 (2)
C1—C4—C5—O1	−9 (2)	C510—C511—C512—C507	−2.1 (19)
C1—C4—C5—O2	179.0 (12)	C508—C507—C512—C511	1.2 (16)
C12—P6—C11—C14	131.1 (9)	P5—C507—C512—C511	−176.1 (9)
C601—P6—C11—C14	12.4 (11)	C11—P6—C601—C606	−108.6 (13)
C607—P6—C11—C14	−108.7 (10)	C12—P6—C601—C606	138.0 (12)
C12—P6—C11—Ir2	−47.1 (7)	C607—P6—C601—C606	13.8 (13)
C601—P6—C11—Ir2	−165.8 (5)	C11—P6—C601—C602	70.5 (10)
C607—P6—C11—Ir2	73.0 (6)	C12—P6—C601—C602	−42.9 (11)
C11—P6—C12—P5	53.4 (8)	C607—P6—C601—C602	−167.0 (9)
C601—P6—C12—P5	174.7 (6)	C606—C601—C602—C603	1.2 (19)
C607—P6—C12—P5	−64.5 (7)	P6—C601—C602—C603	−177.9 (10)
C507—P5—C12—P6	86.9 (7)	C601—C602—C603—C604	−1 (2)
C501—P5—C12—P6	−161.6 (6)	C602—C603—C604—C605	4 (2)
Ir2—P5—C12—P6	−37.7 (7)	C603—C604—C605—C606	−7 (2)
C807—P8—C13—P7	−97.9 (6)	C604—C605—C606—C601	7 (3)
C801—P8—C13—P7	149.9 (5)	C602—C601—C606—C605	−4 (2)
Ir2—P8—C13—P7	28.9 (4)	P6—C601—C606—C605	174.9 (13)
C707—P7—C13—P8	87.1 (6)	C11—P6—C607—C608	21.0 (11)
C701—P7—C13—P8	−159.0 (5)	C12—P6—C607—C608	133.7 (9)
Ir2—P7—C13—P8	−29.2 (4)	C601—P6—C607—C608	−104.0 (10)
P6—C11—C14—C15	−170.9 (9)	C11—P6—C607—C612	−164.6 (8)
Ir2—C11—C14—C15	6.9 (19)	C12—P6—C607—C612	−51.9 (10)
C11—C14—C15—O4	15 (2)	C601—P6—C607—C612	70.4 (10)
C11—C14—C15—O5	−163.9 (12)	C612—C607—C608—C609	5.0 (17)
C107—P1—C101—C106	24.4 (10)	P6—C607—C608—C609	179.2 (9)
C2—P1—C101—C106	134.2 (9)	C607—C608—C609—C610	−4.3 (18)
Ir1—P1—C101—C106	−107.4 (9)	C608—C609—C610—C611	1 (2)
C107—P1—C101—C102	−160.0 (10)	C609—C610—C611—C612	2 (2)
C2—P1—C101—C102	−50.2 (12)	C610—C611—C612—C607	−1.0 (19)
Ir1—P1—C101—C102	68.2 (11)	C608—C607—C612—C611	−2.3 (17)
C106—C101—C102—C103	−0.5 (19)	P6—C607—C612—C611	−176.7 (9)
P1—C101—C102—C103	−176.2 (10)	C707—P7—C701—C706	137.5 (11)
C101—C102—C103—C104	0 (2)	C13—P7—C701—C706	21.4 (12)
C102—C103—C104—C105	2 (2)	Ir2—P7—C701—C706	−86.0 (12)
C103—C104—C105—C106	−2 (2)	P8—P7—C701—C706	−4.9 (16)
C104—C105—C106—C101	1 (2)	C707—P7—C701—C702	−41.5 (10)
C102—C101—C106—C105	0.1 (17)	C13—P7—C701—C702	−157.6 (9)
P1—C101—C106—C105	175.8 (10)	Ir2—P7—C701—C702	95.0 (9)
C101—P1—C107—C108	−136.1 (10)	P8—P7—C701—C702	176.1 (6)
C2—P1—C107—C108	111.9 (10)	C706—C701—C702—C703	1 (2)
Ir1—P1—C107—C108	−5.0 (11)	P7—C701—C702—C703	179.7 (11)
C101—P1—C107—C112	49.5 (10)	C701—C702—C703—C704	2 (2)
C2—P1—C107—C112	−62.5 (10)	C702—C703—C704—C705	−4 (2)
Ir1—P1—C107—C112	−179.4 (7)	C703—C704—C705—C706	2 (2)
C112—C107—C108—C109	−1.1 (17)	C704—C705—C706—C701	2 (2)
P1—C107—C108—C109	−175.5 (10)	C702—C701—C706—C705	−3 (2)
C107—C108—C109—C110	−0.7 (19)	P7—C701—C706—C705	178.3 (10)
C108—C109—C110—C111	2 (2)	C701—P7—C707—C708	94.1 (10)
C109—C110—C111—C112	−1 (2)	C13—P7—C707—C708	−149.0 (9)
C110—C111—C112—C107	−1 (2)	Ir2—P7—C707—C708	−48.4 (10)
C108—C107—C112—C111	1.9 (18)	P8—P7—C707—C708	−105.7 (9)
P1—C107—C112—C111	176.7 (10)	C701—P7—C707—C712	−87.3 (11)
C207—P2—C201—C206	−14.3 (11)	C13—P7—C707—C712	29.5 (11)
C2—P2—C201—C206	−135.3 (10)	Ir2—P7—C707—C712	130.1 (9)
C1—P2—C201—C206	109.1 (10)	P8—P7—C707—C712	72.8 (10)
C207—P2—C201—C202	168.5 (9)	C712—C707—C708—C709	−2.4 (18)
C2—P2—C201—C202	47.5 (11)	P7—C707—C708—C709	176.2 (10)
C1—P2—C201—C202	−68.1 (10)	C707—C708—C709—C710	1 (2)
C206—C201—C202—C203	0.8 (17)	C708—C709—C710—C711	2 (2)
P2—C201—C202—C203	178.0 (9)	C709—C710—C711—C712	−3 (2)
C201—C202—C203—C204	−1 (2)	C710—C711—C712—C707	1 (2)
C202—C203—C204—C205	2 (2)	C708—C707—C712—C711	1.5 (19)
C203—C204—C205—C206	−3 (2)	P7—C707—C712—C711	−177.0 (11)
C202—C201—C206—C205	−1.5 (19)	C807—P8—C801—C802	47.7 (11)
P2—C201—C206—C205	−178.7 (10)	C13—P8—C801—C802	158.9 (10)
C204—C205—C206—C201	2 (2)	Ir2—P8—C801—C802	−97.9 (10)
C2—P2—C207—C212	46.2 (10)	P7—P8—C801—C802	−166.3 (7)
C201—P2—C207—C212	−74.4 (10)	C807—P8—C801—C806	−132.5 (11)
C1—P2—C207—C212	160.3 (9)	C13—P8—C801—C806	−21.3 (12)
C2—P2—C207—C208	−131.5 (9)	Ir2—P8—C801—C806	81.9 (11)
C201—P2—C207—C208	107.9 (9)	P7—P8—C801—C806	13.5 (15)
C1—P2—C207—C208	−17.5 (10)	C806—C801—C802—C803	−1 (2)
C212—C207—C208—C209	2.4 (17)	P8—C801—C802—C803	179.2 (11)
P2—C207—C208—C209	−179.7 (9)	C801—C802—C803—C804	−4 (2)
C207—C208—C209—C210	−4.4 (19)	C802—C803—C804—C805	6 (2)
C208—C209—C210—C211	5 (2)	C803—C804—C805—C806	−4 (2)
C209—C210—C211—C212	−3 (2)	C804—C805—C806—C801	0 (2)
C208—C207—C212—C211	−0.9 (17)	C802—C801—C806—C805	3 (2)
P2—C207—C212—C211	−178.6 (9)	P8—C801—C806—C805	−177.2 (10)
C210—C211—C212—C207	1.2 (19)	C801—P8—C807—C808	−132.9 (11)
C307—P3—C301—C302	40.2 (11)	C13—P8—C807—C808	111.2 (12)
C3—P3—C301—C302	155.9 (10)	Ir2—P8—C807—C808	9.5 (14)
Ir1—P3—C301—C302	−94.7 (10)	P7—P8—C807—C808	67.2 (12)
C307—P3—C301—C306	−139.5 (10)	C801—P8—C807—C812	50.6 (12)
C3—P3—C301—C306	−23.7 (12)	C13—P8—C807—C812	−65.3 (11)
Ir1—P3—C301—C306	85.7 (11)	Ir2—P8—C807—C812	−167.0 (8)
C306—C301—C302—C303	−1 (2)	P7—P8—C807—C812	−109.3 (10)
P3—C301—C302—C303	179.5 (11)	C812—C807—C808—C809	1 (2)
C301—C302—C303—C304	4 (2)	P8—C807—C808—C809	−175.8 (10)
C302—C303—C304—C305	−5 (2)	C807—C808—C809—C810	3 (2)
C303—C304—C305—C306	2 (2)	C808—C809—C810—C811	−5 (3)
C302—C301—C306—C305	−2 (2)	C809—C810—C811—C812	3 (3)
P3—C301—C306—C305	177.1 (10)	C810—C811—C812—C807	1 (2)
C304—C305—C306—C301	2 (2)	C808—C807—C812—C811	−3 (2)
C301—P3—C307—C308	−92.8 (11)	P8—C807—C812—C811	173.7 (13)
C3—P3—C307—C308	149.1 (9)	O1—C5—O2—C6	4 (2)
Ir1—P3—C307—C308	48.7 (11)	C4—C5—O2—C6	176.2 (12)
C301—P3—C307—C312	87.9 (11)	C7—C6—O2—C5	−171.1 (18)
C3—P3—C307—C312	−30.2 (11)	O4—C15—O5—C16	0.4 (18)
Ir1—P3—C307—C312	−130.6 (9)	C14—C15—O5—C16	179.6 (11)
C312—C307—C308—C309	3.1 (18)	C17—C16—O5—C15	−161.7 (12)
P3—C307—C308—C309	−176.2 (10)	O11—S1—C01—F2	−64.2 (13)
C307—C308—C309—C310	−2 (2)	O12—S1—C01—F2	177.8 (11)
C308—C309—C310—C311	2 (3)	O13—S1—C01—F2	58.1 (13)
C309—C310—C311—C312	−3 (3)	O11—S1—C01—F1	173.1 (10)
C310—C311—C312—C307	4 (2)	O12—S1—C01—F1	55.0 (12)
C308—C307—C312—C311	−4 (2)	O13—S1—C01—F1	−64.6 (12)
P3—C307—C312—C311	175.3 (12)	O11—S1—C01—F3	56.2 (12)
C3—P4—C401—C402	30.3 (12)	O12—S1—C01—F3	−61.9 (12)
C407—P4—C401—C402	138.5 (11)	O13—S1—C01—F3	178.5 (11)
Ir1—P4—C401—C402	−73.4 (11)	O24—S2—C02—F5	61 (2)
C3—P4—C401—C406	−151.5 (11)	O25—S2—C02—F5	−177.7 (17)
C407—P4—C401—C406	−43.3 (12)	O26—S2—C02—F5	−59.5 (19)
Ir1—P4—C401—C406	104.8 (11)	O24—S2—C02—F4	−170.7 (15)
C406—C401—C402—C403	−1 (2)	O25—S2—C02—F4	−49.1 (16)
P4—C401—C402—C403	177.3 (10)	O26—S2—C02—F4	69.1 (15)
C401—C402—C403—C404	0 (2)	O24—S2—C02—F6	−63.4 (15)
C402—C403—C404—C405	−3 (2)	O25—S2—C02—F6	58.3 (14)
C403—C404—C405—C406	7 (3)	O26—S2—C02—F6	176.4 (12)
C402—C401—C406—C405	5 (2)	O37—S3—C03—F7	−177.0 (10)
P4—C401—C406—C405	−173.3 (12)	O39—S3—C03—F7	62.5 (12)
C404—C405—C406—C401	−8 (3)	O38—S3—C03—F7	−57.4 (12)
C401—P4—C407—C408	132.7 (10)	O37—S3—C03—F8	59.0 (12)
C3—P4—C407—C408	−113.4 (10)	O39—S3—C03—F8	−61.6 (12)
Ir1—P4—C407—C408	−12.3 (12)	O38—S3—C03—F8	178.5 (11)
C401—P4—C407—C412	−50.8 (10)	O37—S3—C03—F9	−55.3 (12)
C3—P4—C407—C412	63.1 (9)	O39—S3—C03—F9	−175.9 (10)
Ir1—P4—C407—C412	164.2 (7)	O38—S3—C03—F9	64.2 (12)
C412—C407—C408—C409	−0.2 (19)	O43—S4—C04—F11	−64.1 (18)
P4—C407—C408—C409	175.9 (9)	O41—S4—C04—F11	173.3 (15)
C407—C408—C409—C410	0.8 (19)	O42—S4—C04—F11	56.0 (18)
C408—C409—C410—C411	−1 (2)	O43—S4—C04—F10	176.8 (14)
C409—C410—C411—C412	1 (2)	O41—S4—C04—F10	54.2 (17)
C410—C411—C412—C407	0 (2)	O42—S4—C04—F10	−63.1 (16)
C408—C407—C412—C411	−0.2 (19)	O43—S4—C04—F12	61.8 (13)
P4—C407—C412—C411	−176.7 (10)	O41—S4—C04—F12	−60.9 (13)
C507—P5—C501—C502	−27.1 (11)	O42—S4—C04—F12	−178.1 (12)
C12—P5—C501—C502	−137.6 (9)	C22—O21—C20—O20	6 (5)
Ir2—P5—C501—C502	106.8 (9)	C22—O21—C20—C21	−176.7 (18)
C507—P5—C501—C506	158.2 (9)	C20—O21—C22—C23	−88 (3)
C12—P5—C501—C506	47.6 (11)		

### [Bis(diphenylphosphanyl)methane]carbonyl({[(diphenylphosphanyl)methyl]diphenylphosphanylidene}(ethoxyoxoethanylidene)methanylidene- κ2P,C}hydridoiridium(III) bis(trifluoromethanesulfonate)–dichloromethane–ethyl acetate (6/2/3) (4) Hydrogen-bond geometry (Å, º)

**Table:**

*D*—H···*A*	*D*—H	H···*A*	*D*···*A*	*D*—H···*A*
C2—H2*A*···O43	0.98	2.23	3.190 (15)	165
C3—H3*B*···O41^i^	0.98	2.44	3.404 (14)	167
C12—H12*B*···O25^i^	0.98	2.26	3.226 (14)	170
C13—H13*A*···O38^ii^	0.98	2.58	3.538 (15)	167
C13—H13*B*···O26	0.98	2.48	3.446 (15)	169
C16—H16*B*···O20^iii^	0.98	2.48	3.15 (2)	125
C102—H102···O39	0.94	2.47	3.379 (17)	162
C108—H108···O11^i^	0.94	2.57	3.248 (15)	129
C202—H202···O39	0.94	2.57	3.508 (16)	178
C212—H212···O43	0.94	2.55	3.471 (15)	167
C306—H306···O11^i^	0.94	2.53	3.409 (16)	155
C312—H312···O41^i^	0.94	2.46	3.374 (16)	166
C402—H402···O11^i^	0.94	2.52	3.429 (15)	164
C506—H506···O12^i^	0.94	2.50	3.420 (17)	165
C508—H508···O38^ii^	0.94	2.56	3.284 (16)	134
C602—H602···O12^i^	0.94	2.59	3.519 (16)	172
C612—H612···O25^i^	0.94	2.59	3.511 (17)	165
C706—H706···O38^ii^	0.94	2.47	3.327 (16)	151
C712—H712···O26	0.94	2.41	3.335 (18)	167
C806—H806···O38^ii^	0.94	2.52	3.406 (16)	158
C808—H808···O4	0.94	2.48	2.964 (16)	112
C25—H25*B*···O24	0.98	2.53	3.14 (3)	121
C25*A*—H25*D*···O24	0.98	2.19	3.10 (9)	153
C16—H16*B*···O20^iii^	0.98	2.48	3.15 (2)	125

Symmetry codes: (i) *x*+1, *y*, *z*; (ii) *x*, *y*+1, *z*; (iii) −*x*, *y*+1/2, −*z*+3/2.
